# HIV-1 diversity considerations in the application of the Intact Proviral DNA Assay (IPDA)

**DOI:** 10.1038/s41467-020-20442-3

**Published:** 2021-01-08

**Authors:** Natalie N. Kinloch, Yanqin Ren, Winiffer D. Conce Alberto, Winnie Dong, Pragya Khadka, Szu Han Huang, Talia M. Mota, Andrew Wilson, Aniqa Shahid, Don Kirkby, Marianne Harris, Colin Kovacs, Erika Benko, Mario A. Ostrowski, Perla M. Del Rio Estrada, Avery Wimpelberg, Christopher Cannon, W. David Hardy, Lynsay MacLaren, Harris Goldstein, Chanson J. Brumme, Guinevere Q. Lee, Rebecca M. Lynch, Zabrina L. Brumme, R. Brad Jones

**Affiliations:** 1 Faculty of Health Sciences, Simon Fraser University, Burnaby, BC Canada; 2 British Columbia Centre for Excellence in HIV/AIDS, Vancouver, BC Canada; 3 Infectious Diseases Division, Department of Medicine, Weill Cornell Medical College, New York, NY USA; 4 Department of Microbiology, Immunology and Tropical Medicine, George Washington University, Washington, DC USA; 5 Faculty of Medicine, University of British Columbia, Vancouver, BC Canada; 6 Maple Leaf Medical Clinic, Toronto, ON Canada; 7 Department of Medicine, University of Toronto, Toronto, ON Canada; 8 Center for Research in Infectious Diseases, National Institute of Respiratory Diseases, Mexico City, Mexico; 9 Whitman Walker Health, Washington, DC USA; 10 Department of Microbiology and Immunology, Albert Einstein College of Medicine, Bronx, NY USA

**Keywords:** Microbiology techniques, Sequencing, HIV infections, Translational research

## Abstract

The Intact Proviral DNA Assay (IPDA) was developed to address the critical need for a scalable method for intact HIV-1 reservoir quantification. This droplet digital PCR-based assay simultaneously targets two HIV-1 regions to distinguish genomically intact proviruses against a large background of defective ones, and its application has yielded insights into HIV-1 persistence. Reports of assay failures however, attributed to HIV-1 polymorphism, have recently emerged. Here, we describe a diverse North American cohort of people with HIV-1 subtype B, where the IPDA yielded a failure rate of 28% due to viral polymorphism. We further demonstrate that within-host HIV-1 diversity can lead the IPDA to underestimate intact reservoir size, and provide examples of how this phenomenon could lead to erroneous interpretation of clinical trial data. While the IPDA represents a major methodological advance, HIV-1 diversity should be addressed before its widespread adoption as a principal readout in HIV-1 remission trials.

## Abstract (web summary)

The intact proviral DNA assay quantifies the genomically intact HIV reservoir, but assay failure due to HIV-1 polymorphism has been observed. Here, the authors report a 28% failure rate in a cohort of people with HIV-1, and note within-host HIV-1 diversity as a further challenge to IPDA accuracy.

## Introduction

The Intact Proviral DNA Assay (IPDA) was developed to address the critical need for a precise and scalable method to quantify intact HIV-1 proviruses, which represent the main barrier to achieving HIV-1 remission or cure^1^. This duplexed droplet digital PCR (ddPCR) assay simultaneously targets two HIV-1 regions, the Packaging Signal (Ψ) near the 5’ end of the viral genome and the Rev Responsive Element (RRE) within Envelope (*env*), to distinguish genomically intact proviruses against a large background of defective ones. The IPDA requires less time, resources and biological material than the current gold standard for replication-competent HIV-1 reservoir measurement, the Quantitative Viral Outgrowth Assay (QVOA)^2^, while also quantifying the total proviral burden. Application of the assay to increasing numbers of individuals living with HIV-1 has yielded insights into reservoir composition and dynamics^3–6^, and the assay is being recommended for use in clinical trials evaluating HIV-1 remission strategies^4,5,7^.

Recently however, instances of assay failure, attributable to natural HIV-1 polymorphism in primer and/or probe binding regions, have been described^5–7^. While detection failures are expected for any molecular assay targeting a genetically variable pathogen, this rate has varied markedly across studies, ranging from 0% in the initial two reports (0/62^1^ and 0/81^4^) to 12% (6/50) in the most recent publication^7^, where the assay was performed by the same group. A recent multi-cohort study comprising 400 individuals reports an overall 6.3% failure rate^5^; however, because this study includes the above^4^ and other published cohorts with 0% failure rates^8–10^ it can be inferred that the rates for the unpublished datasets are higher. Thus, while the IPDA is undeniably a major methodological advance, further study is needed to delineate the impact of HIV-1 sequence diversity. This is particularly relevant to clinical trials, which would either require an accurate anticipated rate of failure for power calculations, or strategies to address diversity directly, which might include pre-enrollment screening to determine IPDA-detectability of an individual’s virus and/or development of secondary primers and probes.

Here, we aimed to further develop the IPDA by assessing its performance within a diverse cohort of individuals with HIV-1 subtype B from across North America. Our study is unique in a number of ways. First, with the exception of one study that investigated a subset (20%) of assay failures by HIV-1 sequencing^5^, no studies to date have combined IPDA, QVOA, and HIV sequencing to examine the frequency and potential implications of assay failures in detail. We describe an IPDA failure rate of 28% in our cohort, where we used QVOA to confirm that intact proviruses were indeed present in these cases, and where we present the underlying sequence polymorphisms for each case of failure. Second, we illustrate how within-host HIV diversity—specifically, cases where an individual harbors viral variants that are detectable and others that are undetectable by the assay—could lead the IPDA to underestimate intact HIV-1 reservoir size. We further provide examples of how this type of error could negatively impact clinical trial results interpretation. Third, our study provides an assessment of the IPDA by a group other than its original developers (or the associated company Accelevir Diagnostics). This could benefit the field, given that a major advantage of the IPDA is its potential for broad accessibility. Our failure rate of 28% occurred in the context of our efforts to precisely match the reported experimental conditions, with one exception which could have affected of a minority of these cases. Whereas Peluso et al.^4^ state that gates to define positive droplets can be drawn tightly against double-negative droplets as defined in samples from individuals without HIV-1, we demonstrate that fluorescence signal from Ψ-positive templates (labeled with FAM) can bleed into the *env* channel, creating a false double-positive population, if gated in this manner. Thus, we required separation between negative and positive droplet populations in order to consider a result valid. This represents a previously unreported source of error in the IPDA. Taken together, we present an independent assessment of the scope of the challenge of addressing HIV-1 diversity in the IPDA, and—through sequence characterization—contribute to understanding the catalogue of polymorphisms that will need to be addressed with secondary primers and probes, towards improved iterations of this valuable assay.

## Results

### IPDA measurements in a diverse North American cohort

**Fig. 1:**
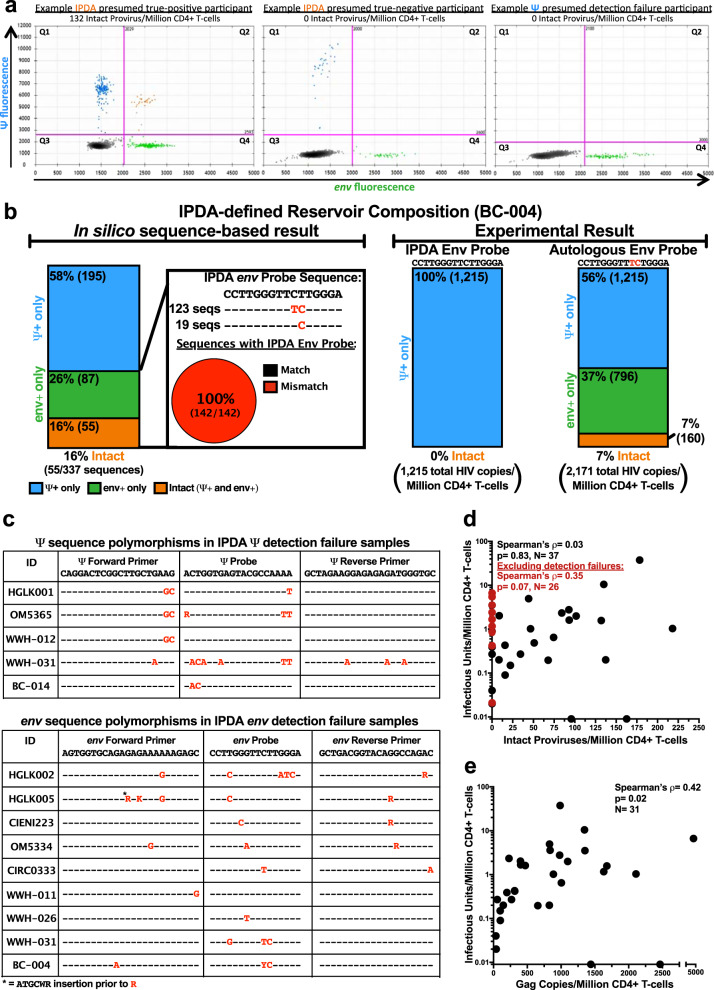
Interindividual HIV diversity can lead to detection failure by the IPDA. **a** IPDA 2D ddPCR plots. Ψ-single-positive (Q1, blue), Ψ- and *env*- double-positive (Q2, orange), double-negative (Q3, gray) and *env* single-positive (Q4, green) events for an IPDA true-positive individual, a presumed IPDA true-negative individual and a presumed case of Ψ detection failure. Plots show merge of four replicate wells. **b** IPDA detection failure due to interindividual HIV diversity. In silico analysis of 337 linked Ψ and *env* probe sequences from proviral DNA of participant BC-004, who originally failed detection in *env*, revealed single nucleotide mismatches (red, inset) to the IPDA *env* probe. These sequences yielded in silico Ψ+(blue), *env*+(green) and intact (Ψ+*env*+; orange) provirus frequencies as shown on left. Experimental results using the published IPDA and autologous *env* probe are shown on right. Source data, including the 337 Ψ and *env* probe sequences and their classifications, are provided in the Source Data File. **c** HIV polymorphisms in IPDA primer and probe region sequences for participants with Ψ or *env* detection failure. Hyphens (**-**) indicate matches to the IPDA primer or probe; red letters indicate mismatches; asterisk indicates insertion location. **d** Correlation between reservoir size as measured by IPDA (Intact Proviruses/Million CD4 + T-cells) and QVOA (Infectious Units/Million CD4 + T-cells). When data from all 37 virally-suppressed participants are included, Spearman’s ρ = 0.03 with a two-tailed p = 0.83. After excluding 11 presumed IPDA detection failures (red datapoints), Spearman’s ρ = 0.35 with a two-tailed p = 0.07. Two individuals for whom no replication-competent viruses were detected (IUPM = 0) are plotted on the *X*-axis. QVOA replicates are detailed the methods; IPDA data represent the point estimate from four merged technical replicates per sample. Source data are provided in the Source Data file. **e** Correlation between QVOA and HIV *gag* copies/million CD4 + T-cells. Analysis comprises 31 unique participants, yielding Spearman’s ρ = 0.42, two-tailed p = 0.02. Two individuals for whom no replication-competent viruses were detected (IUPM = 0) are plotted on the *X*-axis. HIV *gag* copies report the point estimate from at least four merged technical replicates for each sample. Source data are provided in the Source Data File.

We applied the IPDA to 46 individuals with HIV-1 subtype B who had achieved viral suppression from cohorts across Canada, the USA and Mexico. Initial assay results yielded a median of 29 (interquartile range [IQR] 0-93) intact proviruses/million CD4^+^ T-cells (Supplementary Fig. 1); however, among these were 17 participants (37%) for whom the IPDA did not detect any intact (i.e., Ψ and *env* double-positive) proviruses. In four of these individuals, both Ψ- and *env*- single-positive proviruses were detected, suggesting a true-negative result (see example in Fig. 1a and Supplementary Fig. 2). In the remaining 13 individuals, however, the IPDA did not detect Ψ- and/or *env*- single-positive proviruses above background levels and instead yielded ddPCR plots that typify assay failure^5,7^ (Fig. 1a, Supplementary Fig. 2). Specifically, in eight of these participants only Ψ-positive proviruses were detected, in four only *env*-positive proviruses were detected, and in one participant no proviruses were detected. Furthermore, replication-competent HIV-1 was recovered in 11/11 (100%) cases where sufficient biological material was available to perform QVOA, consistent with the failure of IPDA to detect autologous intact proviruses.

### HIV sequence polymorphism can cause IPDA detection failure

We next investigated the presumed cases of IPDA failure using HIV-1 sequencing. Near-full-length single-genome proviral sequencing performed on IPDA *env*-negative participant BC-004 revealed mismatches to the IPDA *env* probe, where in silico predicted reservoir distributions that took these polymorphisms into account (Fig. 1b, left) differed markedly from the original experimentally-obtained result (Fig. 1b, center). Substituting an autologous *env* probe rescued detection to in silico predicted levels (Fig. 1b, right), confirming that HIV-1 polymorphism can cause the IPDA to fail. Mismatches in the probe and/or at the 3′ end of a primer were confirmed in all 13 cases of presumed assay detection failure (Fig. 1c). Together, this yielded an overall failure rate of 13/46 (28%). Indeed, exclusion of these 13 datapoints markedly improved the correlation between IPDA and QVOA results among those for whom sufficient biological material was available to perform the latter assay, from ρ = 0.03, *p* = 0.83 to ρ = 0.35, *p* = 0.07 (Fig. 1d), bringing this correlation more in line with that reported by the original authors^1^ and what is expected biologically. By contrast, the correlation between QVOA and total HIV-1 *gag* DNA, measured using different HIV-1 primers and probe located in a conserved region^11^, was significant without exclusion of these datapoints (Fig. 1e).

### Within-host HIV diversity can cause reservoir underestimation

Although laborious to correct, complete detection failures due to HIV-1 polymorphism are nevertheless easy to flag due to their characteristic ddPCR plot presentation (Fig. 1a, Supplementary Fig. 2). In contrast, within-host HIV-1 diversity in IPDA primer or probe regions (see Fig. 1b for an example of diversity within a probe region) could lead the IPDA to underestimate intact reservoir size if the within-host variants were differentially detectable by the assay. Such partial detection failures would not be easy to identify. Moreover, if IPDA-detectable and nondetectable reservoir subpopulations were differentially susceptible to HIV-1 cure interventions, this could lead to erroneous conclusions regarding intervention efficacy.

**Fig. 2:**
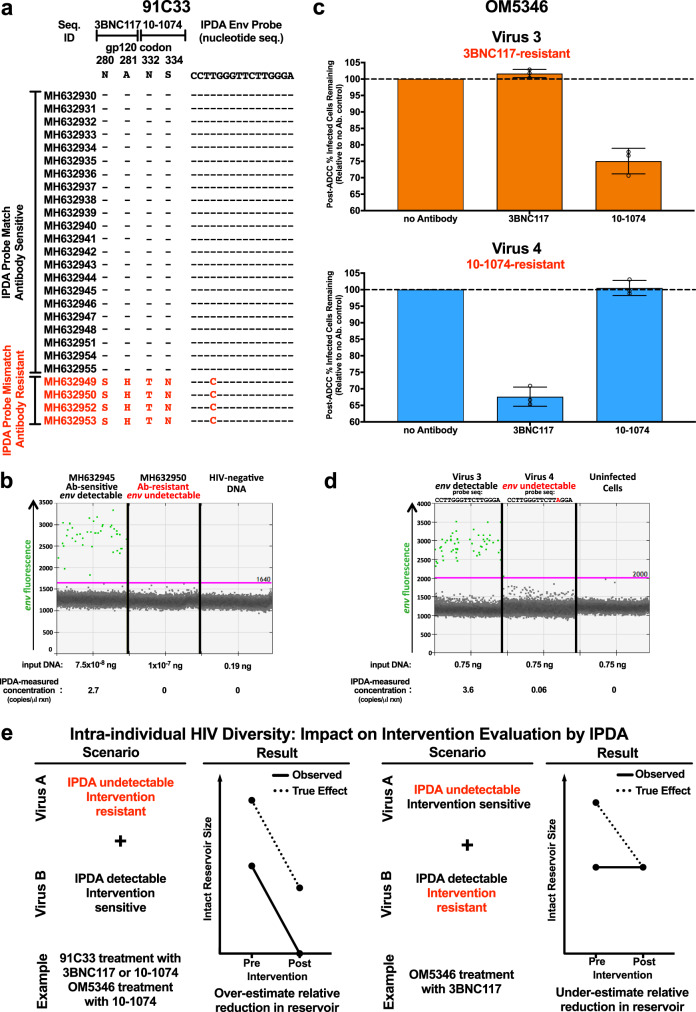
Intraindividual HIV diversity can lead to reservoir underestimation by IPDA. **a** *Env gp120* amino acid and IPDA *env* probe nucleotide sequences from participant 91C33 identified by GenBank accession numbers. 91C33 harbored one HIV subpopulation that matched the IPDA *env* probe and was sensitive to bNAbs 3BNC117 and 10-1074, and another that harbored a mismatch at position 4 of the IPDA *env* probe and was resistant to both bNAbs. Hyphens (**-**) indicate matches to the reference; red letters denote mismatches (IPDA *env* probe) or amino acid substitutions (bNAb). Sequence is abbreviated seq. **b** ddPCR *env* plots from one of 2 independent experiments assessing the ability of the IPDA *env* reaction to detect representative within-host sequences MH632945 (*env* probe match) and MH632950 (*env* probe mismatch), present as purified *env* PCR products of equal length and comparable quantities. Positive droplets are green and negative droplets are grey. See Supplementary Fig. 3 for additional experiments. **c** Virus 3, isolated from participant OM5346’s reservoir, was sensitive to 10-1074-mediated ADCC but resistant to 3BNC117; vice-versa for virus 4. Box and error bars indicate mean and standard deviation of three technical replicates, from one of two independent experiments. Open circles denote individual datapoints. Source data are provided in the Source Data File. **d** 1D IPDA *env* ddPCR plots from one of 2 independent experiments for cells infected in vitro with virus 3 (IPDA *env* probe match) or virus 4 (G13A mismatch). Both viruses were detectable using an alternative primer/probe set (Supplementary Fig. 6). **e** Possible impacts of intraindividual HIV diversity on IPDA-measured reservoir changes following a hypothetical intervention. The solid line indicates the observed effect by IPDA; dashed line indicates the true effect. An intervention’s impact on the reservoir could be overestimated when a subset of within-host HIV sequences are undetectable by IPDA and resistant to treatment (e.g., 91C33 during treatment with 3BNC117 or 10-1074, or OM5346 during treatment with 10-1074). Conversely, an intervention’s impact on the reservoir could be underestimated when a subset of within-host HIV sequences are undetectable by IPDA and sensitive to treatment (e.g., OM5346 during treatment with 3BNC117). In a worst-case scenario, one could erroneously conclude that the treatment had no effect.

Although such a scenario remains hypothetical, we nevertheless illustrate our point using HIV-1-specific broadly neutralizing antibodies (bNAbs) as an example. bNAbs can facilitate elimination of HIV-1-infected cells^12^ in part by targeting them for antibody-dependent cellular cytotoxicity (ADCC)^12,13^. As such, they are being evaluated in clinical trials, some of which use the IPDA as a readout (e.g., ACTG A5386^14^). Participant 91C33 from a published trial^15^ provides a hypothetical example. This individual did not respond to (off-ART) infusions of the bNAbs 3BNC117 and 10-1074 because they harbored a plasma HIV-1 subpopulation that was resistant to both bNAbs^15^ (Fig. 2a). Of note, this plasma HIV-1 subpopulation also harbored a mismatch to the IPDA *env* probe that we predicted would cause signal failure. We experimentally confirmed that, using the published conditions, the IPDA could not detect templates harboring this polymorphism (Fig. 2b), though signal could be detected if the annealing/extension temperature was lowered to 53 °C to account for the mismatch (Supplementary Fig. 3). In contrast, templates representing the bNAb-sensitive strains could be detected readily using the published conditions (Fig. 2b). Theoretically, if a person harboring such diversity in their reservoir were to be successfully treated with one or both bNAbs in a trial that used the IPDA as the sole readout, the intervention’s effect on reducing the reservoir would be overestimated because the assay was only capable of detecting the susceptible reservoir fraction (Fig. 2e, left).

ART-suppressed participant OM5346 provides another example. Pre-ART HIV-1 drug resistance genotyping identified subtype B infection; however, single-genome sequencing of pre-ART plasma and proviruses sampled during long-term ART revealed co-infection with a non-B strain (Supplementary Fig. 4). While the IPDA is only designed for subtype B, the co-infecting strain harbored no critical mismatches within IPDA target regions except for a G-to-A mismatch at position 13 (G13A) of the *env* probe. Importantly, G13A is amongst the most frequent *env* probe polymorphisms in subtype B (and was observed in 2/10 study participants for whom *env* was sequenced), and the original report indicated that the IPDA was capable of detecting env sequences harboring it as the sole variant, at least when present on a plasmid template^1^. As such, the assay should be able to detect this strain despite its non-B status. Using QVOA, we successfully isolated replication-competent subtype B (virus 3) and co-infecting (virus 4) strains from OM5346’s reservoir, confirming both of these as true intended targets of the IPDA (Fig. 2c). We further observed that these viruses were differentially susceptible to 3BNC117- and 10-1074-mediated ADCC: while virus 3-infected cells could be eliminated by 10-1074-, but not 3BNC117-, mediated ADCC (25% and 0% relative reduction, respectively), the opposite was true for virus 4 (0% and 32% relative reduction, respectively) (Fig. 2c, Supplementary Fig. 5). We further confirmed that, while the IPDA could readily detect provirus 3 at concentrations approximating biological samples, the assay could not distinguish provirus 4 at these concentrations (Fig. 2d, Supplementary Fig. 6a). Similarly, at biological concentrations, the IPDA could not detect a synthetic DNA template encoding the virus 4 sequence, though noticeable signal elevation occurred at ~100-fold higher template concentrations (Supplementary Fig. 6b). Importantly, a secondary *env* primer/probe set (see below and methods) readily detected both viruses at all concentrations, regardless of template type (Supplementary Fig. 6). In the hypothetical case where this individual were to be successfully treated with a bNAb in a trial that used IPDA as a sole readout, different erroneous interpretations could occur depending on the intervention used. Specifically, if this individual were to be successfully treated with 10-1074, the IPDA would overestimate the intervention’s effect on the reservoir because the assay was only capable of detecting the susceptible reservoir fraction. In contrast, if the individual were to be successfully treated with 3BNC117, the IPDA would erroneously conclude that the intervention had no effect because the assay was only capable of detecting the resistant reservoir fraction (Fig. 2e, right).

### Optical bleed-through as a source of error in the IPDA

**Fig. 3:**
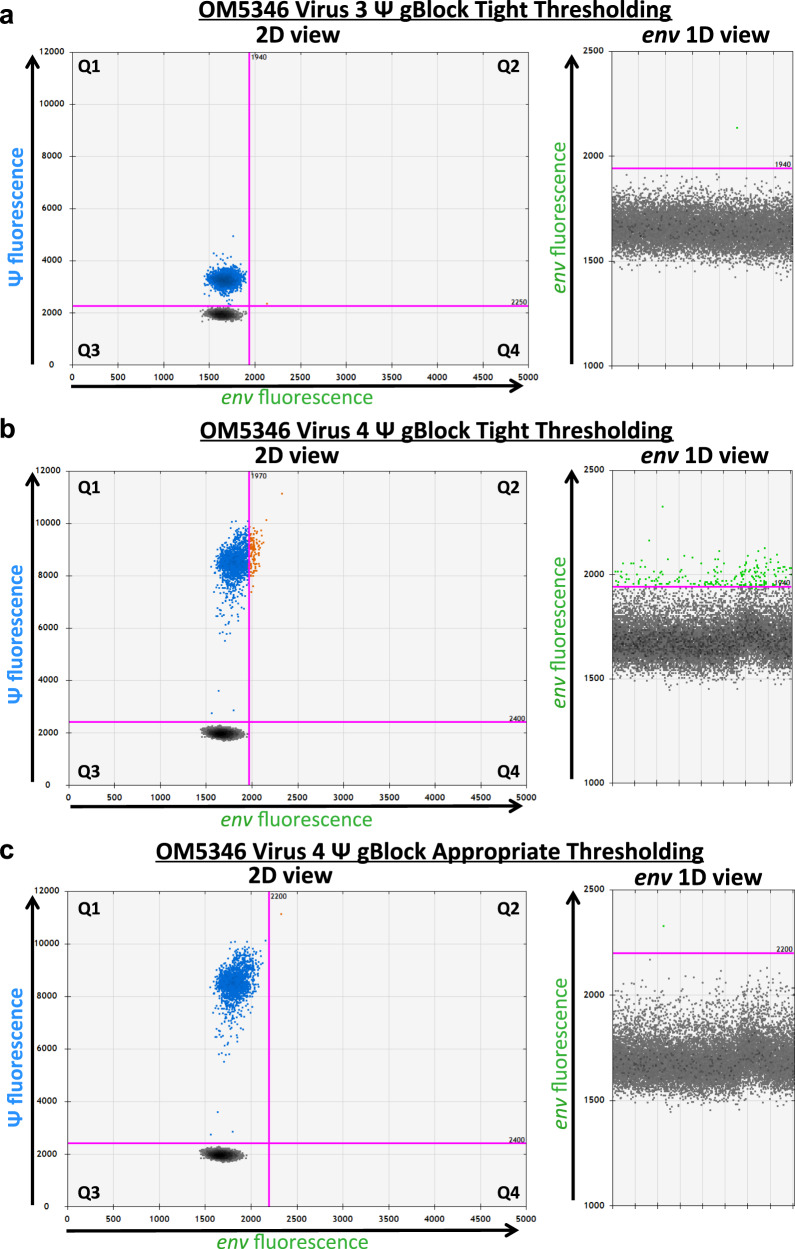
HIV sequence-specific optical bleed-through prohibits tight thresholding on negative populations. **a** 2D and *env* 1D plots of OM5346 virus 3 Ψ region sequence when tested as a synthetic DNA gene fragment (Virus 3 Ψ gBlock) without corresponding *env* template. Minimal Ψ- to *env*- channel spillover occurs and the positive droplet threshold can be drawn tightly to the double-negative population without consequence (note the presence of one false double-positive droplet). **b** 2D and *env* 1D plots of OM5346 virus 4 Ψ region sequence, tested as a synthetic DNA gene fragment (Virus 4 Ψ gBlock), without corresponding *env* template. Drawing a tight threshold causes optical bleed-through of Ψ (FAM) fluorescence into the *env* (VIC) channel to yield a false-positive *env* (and by extension, false-positive intact) signal. **c** 2D and *env* 1D plots of OM5346 Virus 4 Ψ region sequence, tested as a synthetic DNA gene fragment (Virus 4 Ψ gBlock) without corresponding *env* template, with a threshold drawn at an appropriate distance from the double-negative population. This threshold accommodates the fluorescence shift and thus avoids the creation of false-positive intact or *env*-positive droplet population (note the presence of a single false double-positive droplet). Representative plots from one of three technical replicates from one experiment are shown.

Although HIV-1 polymorphism can clearly cause the IPDA to fail in some instances, certain mismatches can be tolerated^4,5^. This is due to the assay’s use of endpoint (rather than real-time) PCR detection, such that polymorphisms that reduce signal can yield lower-amplitude droplets that can nevertheless still be scored as positive, provided that they are sufficiently separated from the negative population. In fact, participant OM5346’s two viral populations illustrate this phenomenon: virus 3, which harbors a single nucleotide deletion as well as a mismatch in the Ψ probe, produces modest yet clearly discernible signal in the Ψ channel, whereas virus 4, which also harbors minor primer/probe mismatches, produces higher-amplitude Ψ signal (Fig. 3). Detailed investigation of such instances of HIV-1 sequence-driven amplitude modulations led us to identify another source of potential error, in the form of optical bleed-through, a challenge in multiplexed digital PCR assays^16^. This occurs when fluorescence from the Ψ channel, which features the FAM fluorophore, bleeds into the *env* channel, which features the VIC fluorophore, to varying extents. This in turn has implications for the appropriate placement of thresholds defining negative and positive populations. Critically, because the extent of bleed-through is determined by the amplitude of the Ψ signal, which is in turn determined by the extent of match to the assay probe, the extent of fluorescence spillover is HIV-1 sequence-specific. We demonstrate this by applying the IPDA to synthetic templates encoding the Ψ regions of OM5346 viruses 3 and 4, without a corresponding *env* template present (Fig. 3). Virus 3′s Ψ signal, which is modest, does not bleed into the *env* channel. By contrast, virus 4′s high amplitude Ψ signal bleeds noticeably into the *env* channel. Thus, whereas a tight threshold based on the negative population can be drawn for virus 3, doing so for virus 4 creates *env* signal (and in fact intact provirus signal) even when no *env* template is present. This Ψ channel bleed-through for virus 4 thus likely contributed to the small amount of *env* signal in Fig. 2d (events adjoining the negative population). While ddPCR instruments are factory-tuned to mitigate spectral overlap, this basic property of fluorophores underscores the instrument manufacturer’s instruction that appropriate thresholds can only be drawn if negative and positive populations clearly separate from one another^17^. Similarly, it cautions against the setting of gates solely based on data from uninfected donors, as suggested by Peluso et al.^4^ as a strategy for suboptimal amplifications.

### Addressing interhost HIV diversity in the IPDA

**Fig. 4:**
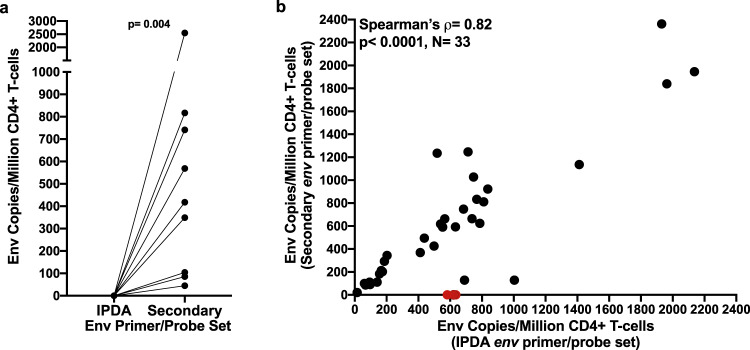
Performance of the secondary *env* primer/probe set. **a** A secondary *env* primer/probe set rescued detection in all 9 cases of IPDA *env* detection failure (*p* = 0.004, two-sided Wilcoxon signed-rank test). **b** A secondary *env* primer/probe set performs comparably to the IPDA in samples detectable by the latter assay. Black datapoints denote 33 participants whose reservoir was detectable by both IPDA and secondary *env* primer/probe sets; red datapoints denote three participants whose reservoir was detectable by IPDA but not using the secondary *env* primer/probe set. Excluding the latter three points yielded a Spearman’s ρ = 0.82, *p* < 0.0001 (shown on figure). Including these three red datapoints yielded a Spearman’s ρ = 0.68, *p* < 0.0001. Datapoints show point estimate from four merged technical replicates for each participant sample. Source data are provided in the Source Data File.

The IPDA offers major scalability and accessibility advantages over existing molecular^18^ or culture-based approaches^2^, and a recent study that employed a quadruplexed qPCR assay confirmed that, of all probes evaluated, those used in the IPDA offered the greatest selectivity for intact proviruses^18^. Unfortunately, the requirement to discriminate proviral defects in the Ψ region restricts the options for placement and sequence of these oligonucleotides^19^, and the limited data currently available for Ψ region polymorphisms in IPDA failure cases (Fig. 1c and ref. ^5^) reveal few common polymorphisms that could be used to design secondary primer/probe sets. By contrast, placement of the 3′ target is less constrained^1^. This allowed us to develop a secondary primer/probe set in the intact-discriminating RRE region, approximately 50 bases downstream of the original location, as a first step towards addressing HIV-1 diversity with a strategy other than autologous oligonucleotide design^3,5^ or data exclusion^7^. This primer/probe set rescued detection of *env*-positive proviruses in 9/9 participants with IPDA *env* detection failure (Fig. 4a). When applied to the 36 participants for whom the IPDA detected *env*-positive proviruses (excluding OM5346 who harbored within-host diversity), it failed to detect *env*-positive proviruses in 3 (8%) individuals, indicating that it is not a universal solution. For the remaining 33 (92%), however, it yielded measurements that correlated strongly with those from the IPDA (Fig. 4b). The secondary primer/probe set could therefore be used to confirm instances of detection failure without confirmatory viral sequencing as well as to estimate intact provirus levels, though users must note that, unlike the IPDA *env* reaction, it cannot discriminate hypermutated sequences.

## Discussion

The IPDA is undeniably a major advance in HIV-1 persistence research, but recent reports of variable and increasing failure rates due to HIV-1 polymorphism are concerning. While the original and early follow-up studies acknowledged HIV-1 diversity as a potential limitation that would require custom primer/probe sets, neither study reported any failures due to viral polymorphism (out of 62 and 81 participants, respectively)^1,4^. More recent studies of 61 and 50 participants reported failure rates of 9.8% and 12%, respectively^6,7^. The largest study undertaken to date using the IPDA, a multi-cohort study published by the assay’s developers, reports an overall failure rate of 6.3% of 400 participants^5^, although it is important to note that this study includes a number of published cohorts including four with 0% failure rates^4,8–10^. As such, the inferred failure rate for some of the unpublished datasets could be as high as 18%, a value that approaches the 28% rate reported in the present study.

Taken together, our findings indicate that as the IPDA is applied to diverse cohorts, detection failures will occur, and not infrequently. The data also together suggest that failure rates may vary markedly by cohort, presumably due to local HIV-1 diversity in regions where HIV-1 subtype B circulates. Indeed, failure rates differed markedly between regions in our own study: whereas the failure rates in the Vancouver and Toronto cohorts were 2/15 (13%) and 3/19 (16%) respectively, those in the Washington, D.C. and Bronx, N.Y. cohorts were 4/7 (57%) and 3/4 (75%) respectively. Moreover, these failures were not attributable to shared HIV-1 polymorphisms nor to epidemiologic linkage (Fig. 1c), and all infections were HIV-1 subtype B.

The first step towards mitigating this issue is to raise awareness: samples that yield no Ψ or *env* single-positive proviruses should be flagged as unreportable until HIV-1 polymorphism has been addressed (e.g., using autologous primers/probes^1,4^). Nevertheless, elevated failure rates limit the assay’s scalability and utility: autologous probe design is time-consuming and costly, and exclusion of assay failures could bias study results if failure rates are substantial. Our findings therefore underscore the importance of better understanding the frequency and nature of HIV-1 polymorphisms within IPDA target regions and their impact on intact proviral quantification. Analysis of HIV-1 subtype B sequences from unique individuals in the Los Alamos HIV database revealed that 23% of 9360 *env* sequences harbored at least one *env* probe mismatch (which is similar to our *env* detection failure rate of 9/46 or 20%), while 50% of 1489 sequences harbored at least one Ψ probe mismatch (which is substantially greater than our Ψ detection failure rate of 5/46 or 11%). This suggests that the *env* reaction may be more sensitive to polymorphism than the Ψ reaction, but larger studies linking proviral sequence to IPDA readouts are needed to confirm this.

Similarly, due to a paucity of studies that pair detailed within-host sequence characterization with IPDA measurements, it is not currently possible to estimate the extent and impact of within-host HIV-1 diversity on intact reservoir quantification by the IPDA. Nevertheless, when taken together with Gaebler et al.’s^18^ observation that four of nine (44%) studied reservoirs were heterogeneous in an IPDA probe region, our combined observations suggest that the impact of within-host HIV-1 diversity on IPDA accuracy may be non-negligible.

In conclusion, we provide an independent assessment of HIV-1 sequence diversity as a source of error in the IPDA by integrating IPDA, QVOA, and viral sequence data to characterize detection failures. We report a substantially higher rate of complete detection failures than previous studies, and additionally identify partial detection failures, attributable to within-host viral variation, as a source of error in the IPDA. The latter issue is particularly challenging given that it is essentially impossible to detect without HIV-1 sequencing, but given the reservoir’s highly dynamic nature^20^ and our identification of cases where within-host diversity is linked to HIV-1 cure intervention susceptibility, it should not be ignored. Given the clear value of the IPDA, iterative efforts to refine this assay are a priority. In the meantime, we recommend that proviral sequencing, at minimum within the IPDA target regions, should be performed for participants of HIV-1 remission clinical trials in which the IPDA is employed, and secondary and/or autologous primers/probes used to mitigate HIV-1 diversity.

## Methods

### Participants and ethics statement

We studied 46 individuals with HIV-1 subtype B who were recruited to cohorts in Toronto (*N* = 19), Vancouver (*N* = 15), New York City (*N* = 4), Washington, D.C. (*N* = 7), and Mexico City (*N* = 1). For Vancouver participants, peripheral blood mononuclear cells (PBMCs) were isolated by density gradient separation and cryopreserved (−150 °C, 90% Fetal Bovine Serum+ 10% DMSO). Participants recruited at other sites provided a leukapheresis sample from which PBMC were isolated and cryopreserved as above. All participants were on long-term, virally suppressive combination antiretroviral therapy (cART) at time of sampling, with the exception of one elite controller. Participants’ duration of untreated HIV-1 infection ranged from 1 month to >10 years, though the exact duration was unknown for most participants. Ethical approval to conduct this study was obtained from the Institutional Review Boards of Simon Fraser University, Providence Health Care/University of British Columbia, Weill Cornell Medicine, and the George Washington University. All participants provided written informed consent.

### Quantitative Viral Outgrowth Assay (QVOA)

For participants for whom sufficient biological material was available, the Quantitative Viral Outgrowth Assay (QVOA) was performed^2^. CD4 + T-cells were isolated from PBMCs by negative selection and plated in serial dilution at either 4 or 6 concentrations (12 replicate wells/concentration, 24-well plates). CD4 + T-cells were stimulated with phytohemagglutinin (PHA, 2 μg/mL) and irradiated allogeneic HIV-1-negative PBMCs were added to further induce viral reactivation. MOLT-4/CCR5 cells were added at 24-h post-stimulation as targets for viral infection. Culture media (RPMI 1640 + 10% FBS + 1% Pen/Strep +50 U/mL IL-2 + 10 ng/mL IL-15) was changed every 3 days and p24 enzyme-linked immunosorbent assay (ELISA) was run on day 14 to identify virus-positive wells. Infectious Units per Million CD4 + T-cells (IUPM) was determined using the Extreme Limiting Dilution Analysis (ELDA) software (version 1.0, http://bioinf.wehi.edu.au/software/elda/)^21^. Culture supernatants from virus-positive wells were frozen (−80 °C) for future use.

### Intact Proviral DNA Assay (IPDA)

Genomic DNA was isolated from a median 4.5 (interquartile range [IQR] 4-5) million CD4 + T-cells using the QIAamp DNA Mini Kit (Qiagen) with precautions to minimize DNA shearing. Intact HIV-1 copies/million CD4 + T-cells were determined by droplet digital PCR (ddPCR) using the Intact Proviral DNA Assay (IPDA)^1^, where HIV-1 and human RPP30 reactions were conducted independently in parallel and copies were normalized to the quantity of input DNA. In each ddPCR reaction, a median 7.5 ng (IQR 7– 7.5 ng) (RPP30) or a median 750 ng (IQR 700– 750 ng) (HIV-1) of genomic DNA was combined with ddPCR Supermix for Probes (no dUTPs, BioRad), primers (final concentration 900 nM, Integrated DNA Technologies), probe(s) (final concentration 250 nM, ThermoFisher Scientific) and nuclease free water. Primer and probe sequences (5′–>3′) were: RPP30 Forward Primer- GATTTGGACCTGCGAGCG, RPP30 Probe- VIC-CTGACCTGAAGGCTCT- MGBNFQ, RPP30 Reverse Primer- GCGGCTGTCTCCACAAGT; RPP30-Shear Forward Primer- CCATTTGCTGCTCCTTGGG, RPP30-Shear Probe- FAM- AAGGAGCAAGGTTCTATTGTAG- MGBNFQ, RPP30-Shear Reverse Primer- CATGCAAAGGAGGAAGCCG; HIV-1 Ψ Forward Primer- CAGGACTCGGCTTGCTGAAG, HIV-1 Ψ Probe- FAM- TTTTGGCGTACTCACCAGT- MGBNFQ, HIV-1 Ψ Reverse Primer- GCACCCATCTCTCTCCTTCTAGC; HIV-1 *env* Forward Primer- AGTGGTGCAGAGAGAAAAAAGAGC, HIV-1 *env* Probe- VIC-CCTTGGGTTCTTGGGA- MGBNFQ, anti-Hypermutant *env* Probe- CCTTAGGTTCTTAGGAGC- MGBNFQ, HIV-1 *env* Reverse Primer- GTCTGGCCTGTACCGTCAGC. For participant BC-004, an autologous *env* probe was designed by modifying the published IPDA *env* probe to match the participant’s sequence (VIC-CCTTGGGTTTCTGGGA- MGBNFQ). Droplets were prepared using either the Automated or QX200 Droplet Generator (BioRad) and cycled at 95 °C for 10 min; 45 cycles of (94 °C for 30 sec, 59 °C for 1 min) and 98 °C for 10 min^1^. Droplets were analyzed on a QX200 Droplet Reader (BioRad) using QuantaSoft software (BioRad, version 1.7.4), where replicate wells were merged prior to analysis. Four technical replicates were performed for each participant sample, where a median (IQR) 243,100 (106,200–265,950) cells were assayed in total. Intact HIV-1 copies (Ψ and *env* double-positive droplets) were corrected for DNA shearing based on the frequency of RPP30 and RPP30-Shear double-positive droplets. The median (IQR) DNA shearing index (DSI), measuring the proportion of sheared DNA in a sample, was 0.31 (0.28–0.35), highly comparable to that reported by the original authors^1^. For the experiments that evaluated the IPDA’s ability to detect the specific *env* sequences harbored by participants 91C33 (Fig. 2b, Supplementary Fig. 3) and OM5346 (Supplementary Fig. 6b), synthetic templates (purified PCR amplicons for 91C33 and commercially-synthesized gBlocks [IDT] for OM5346) were used as targets. Total HIV-1 *gag* copies/million CD4 + T-cells^11^ (Fig. 1e) were measured in reactions prepared using RPP30, as described above, and the following HIV-1-specific primers and probe (5′–>3′): HIV-1 *gag* Forward—TCTCGACGCAGGACTCG; HIV-1 *gag* Reverse−TACTGACGCTCTCGCACC; HIV-1 *gag* Probe−FAM−CTCTCTCCT/ZEN/TCTAGCCTC- 3IABkFQ (IDT). Reactions were cycled at: 95 °C for 10 min; 40 cycles of (94 °C for 30 sec, 53 °C for 1 min) and 98 °C for 10 min. A minimum of four (maximum eight) technical replicates were performed for each sample, where data from replicate wells was merged prior to analysis.

### HIV-1 proviral amplification and sequencing

Single-template, near-full-length proviral amplification was performed on DNA extracted from CD4 + T-cells by nested PCR using Platinum Taq DNA Polymerase High Fidelity (Invitrogen) such that ~25% of the resulting PCR reactions yielded an amplicon. First round primers were: Forward−AAATCTCTAGCAGTGGCGCCCGAACAG, Reverse−TGAGGGATCTCTAGTTACCAGAGTC. Second round primers were: Forward—GCGCCCGAACAGGGACYTGAAARCGAAAG, Reverse−GCACTCAAGGCAAGCTTTATTGAGGCTTA. Reactions were cycled as follows: 92 °C for 2 min; 10 cycles of (92 °C for 10 sec, 60 °C for 30 sec, and 68 °C for 10 min); 20 cycles of (92 °C for 10 sec, 55 °C for 30 sec, and 68 °C for 10 min); 68 °C for 10 min^22,23^. Amplicons were sequenced using Illumina MiSeq technology and de novo assembled using the custom software MiCall (https://github.com/cfe-lab/MiCall) which features an in-house modification of the Iterative Virus Assembler (IVA)^24^, or through collaboration with the Massachusetts General Hospital CCIB Core.

### In silico predicted IPDA results

Linked Ψ and *env* probe region sequences were excised from BC-004 proviral sequences for in silico prediction of IPDA results under the assumption that intact probe regions would be detected by the assay. A Ψ or *env* probe region sequence was considered defective if it contained mutations associated with defective proviruses (e.g., those consistent with hypermutation^1^ or common splice donor site mutations^19^) or if it was completely absent. All other probe region sequences, regardless of whether they matched the published IPDA probe sequence, were considered intact.

### Targeted HIV-1 sequencing

For participant OM5346, HIV-1 RNA was extracted from QVOA outgrowth viruses and pre-ART plasma, after which gp160 (QVOA) and gp120/Pol (plasma) were amplified from endpoint-diluted templates by nested RT-PCR using HIV-1-specific primers and high fidelity enzymes. For participants with suspected IPDA detection failure, and for whom HIV-1 sequences were not already available, the IPDA Ψ and/or *env* amplicon regions were bulk-amplified from extracted proviral DNA using HIV-1-specific primers. Amplicons were sequenced using either Sanger (3730xl, Applied BioSystems) or Next Generation (Illumina MiSeq) technologies. Sanger chromatograms were analyzed using Sequencher (version 5.0.1, Gene Codes), while Illumina MiSeq reads were de novo assembled as described above.

### Antibody-dependent cellular cytotoxicity (ADCC) assays

Total CD4 + T-cells were isolated, as described above, from HIV-1-negative donors and activated with anti-CD3 (Biolegend, catalogue number: 317326, clone: OKT3, 1:1000 dilution) and anti-CD28 antibodies (Biolegend, catalogue number: 302934, clone: CD28.2, 1:1000 dilution). Cells were then infected with the participant virus of interest collected from QVOA supernatant, and monitored by flow cytometry by intracellular staining for HIV-1-Gag (Beckman Coulter, catalogue number: 6604665, clone: KC57-RD1, 1:100 dilution) until cells were >5% Gag +. CD4 + T-cells were washed and incubated with the broadly neutralizing antibody of interest (either 3BNC117 or 10-1074, NIH AIDS Reagent Program, catalogue numbers: 12474 and 12477, respectively, 10 μg/mL) for 2 h. Natural Killer (NK) cells were negatively selected (EasySep, StemCell Technologies) from PBMCs of allogeneic, HIV-1-negative donors and activated using interleukin-15 (IL-15) provided by the National Cancer Institute Biological Research Branch. Activated NK cells were then co-cultured with the infected, antibody-treated CD4 + T-cells for 16 h at an effector-to-target (E:T) ratio of 1:1. Following co-culture, cells were stained with fluorophore-conjugated antibodies against human IgG (Southern BioTech, Catalogue number: 2040-31, 1:200 dilution), CD3 (Biolegend, catalogue number: 344842, clone SK7, 1:200 dilution), CD56 (Biolegend, catalogue number: 318334, clone: HCD56, 1:200 dilution), and CD4 (Biolegend, catalogue number: 200521, clone: RPA-T4, 1:200 dilution), as well as intracellular Gag (as above) and LIVE/DEAD Fixable Aqua Stain amine-reactive dye (Invitrogen). The percentages (%) of infected cells remaining post-ADCC relative to no antibody control were then determined using the following formula: (1 − ((%Gag + cells among viable CD3 + cells in no antibody condition) − (%Gag + cells among viable CD3 + cells in test condition))/(%Gag + cells amongst viable CD3 + cells under no antibody condition)) × 100. Two independent experiments were conducted, each with three technical replicates. Data were collected using Attune^TM^ NxT software (ThermoFisher Scientific, version 2.7) and analyzed using FlowJo (Becton Dickinson, version 10.6).

### Within-host HIV-1 phylogenetic analysis

For participant OM5346, single-genome HIV-1 amplification and sequencing was performed from pre-ART plasma (sampled in 2012), proviruses from purified CD4 + T-cells isolated during suppressive cART (sampled in 2017 and 2019), and replication-competent HIV-1 strains isolated by QVOA (from the 2017 sample) as described above. The 2012 and 2017/2019 samples were shipped directly to, and amplified in, two separate laboratories. A plasma HIV-1 *pol* sequence derived from clinical drug resistance testing in 2012 was also incorporated into the analysis. *pol* and *gp120* sequences were specifically amplified or retrieved from near-full genome proviral sequences using GeneCutter (version 1, https://www.hiv.lanl.gov/content/sequence/GENE_CUTTER/cutter.html). Sequences were multiply-aligned using MAFFT (version 7.427)^25^ in a codon-aware manner and inspected in AliView (version 1.19)^26^. Maximum-likelihood phylogenies were inferred using PhyML (version 3.0)^27^ under a General Time Reversible nucleotide substitution model and visualized using FigTree (version 1.3.1).

### Design of secondary *env* ddPCR primer/probe set

HIV-1 Subtype B *envelope* sequences, limited to one per individual, were retrieved from the HIV LANL database (*N* = 4670, https://www.hiv.lanl.gov/components/sequence/HIV/search/search.html) and aligned. Sequence conservation was assessed across 16–25 base-pair intervals, and three intervals that maximized sequence conservation while meeting ddPCR assay specifications (i.e., amplicon length, T_m_) were chosen as secondary primer and probe locations. These were: Secondary *env* Forward Primer ACTATGGGCGCAGCGTC (representing nucleotides 7809–7825 in the HIV-1 genomic reference HXB2; predicted sequence conservation 79%), Secondary *env* Probe VIC-CTGGCCTGTACCGTCAG- MGBNFQ (HXB2 nucleotides 7833–7849, 83% conservation), Secondary *env* Reverse Primer CCCCAGACTGTGAGTTGCA (HXB2 nucleotides 7939–7921, 88% conservation). Reaction composition and cycling conditions were same as those used for the IPDA as described above. Accurate quantification using the Secondary *env* primer/probe set was verified using DNA extracted from the J-Lat 9.2 cell line (obtained from the NIH AIDS Reagent Program, Division of AIDS, NIAID, NIH, contributed by Dr. Eric Verdin)^28^ (Supplementary Fig. 7), where three independent experiments, each with four technical replicates, were performed and replicate wells were merged prior to data analysis.

### Statistical analyses

All statistical analyses were performed using GraphPad Prism (version 8). All statistical tests were two-sided.

### Reporting summary

Further information on research design is available in the Nature Research Reporting Summary linked to this article.

## Supplementary information

Supplementary Information

Reporting Summary

## Data Availability

All data that support the findings of this study are available in the manuscript, Figures and Supplementary Figures. HIV sequences used for phylogenetic inference are available in GenBank under accession numbers MT792083-MT792233; HIV sequences for participant 91C33 were previously published under GenBank accession numbers MH632930- MH632955^15^. HIV-1 subtype B sequences used to identify the location for the secondary *env* primer/probe set are available through the Los Alamos National Laboratory HIV sequence database (https://www.hiv.lanl.gov/components/sequence/HIV/search/search.html). Additional information is available from the corresponding authors upon request. Source data are provided with this paper.

## Source data

Source Data

## Code availability

Code for the custom software MiCall is available at https://github.com/cfe-lab/MiCall.
