# Author Correction: HIV-1 diversity considerations in the application of the Intact Proviral DNA Assay (IPDA)

**DOI:** 10.1038/s41467-021-23515-z

**Published:** 2021-05-13

**Authors:** Natalie N. Kinloch, Yanqin Ren, Winiffer D. Conce Alberto, Winnie Dong, Pragya Khadka, Szu Han Huang, Talia M. Mota, Andrew Wilson, Aniqa Shahid, Don Kirkby, Marianne Harris, Colin Kovacs, Erika Benko, Mario A. Ostrowski, Perla M. Del Rio Estrada, Avery Wimpelberg, Christopher Cannon, W. David Hardy, Lynsay MacLaren, Harris Goldstein, Chanson J. Brumme, Guinevere Q. Lee, Rebecca M. Lynch, Zabrina L. Brumme, R. Brad Jones

**Affiliations:** 1 Faculty of Health Sciences, Simon Fraser University, Burnaby, BC Canada; 2 British Columbia Centre for Excellence in HIV/AIDS, Vancouver, BC Canada; 3 Infectious Diseases Division, Department of Medicine, Weill Cornell Medical College, New York, NY USA; 4 Department of Microbiology, Immunology and Tropical Medicine, George Washington University, Washington, USA; 5 Faculty of Medicine, University of British Columbia, Vancouver, BC Canada; 6 Maple Leaf Medical Clinic, Toronto, ON Canada; 7 Department of Medicine, University of Toronto, Toronto, ON Canada; 8 Center for Research in Infectious Diseases, National Institute of Respiratory Diseases, Mexico City, Mexico; 9 Whitman Walker Health, Washington, DC, USA; 10 Department of Microbiology and Immunology, Albert Einstein College of Medicine, Bronx, NY USA

**Keywords:** Microbiology techniques, Sequencing, HIV infections, Translational research

Correction to: *Nature Communications* 10.1038/s41467-020-20442-3, published online 08 January 2021.

The original version of this Article contained an error for the ‘RPP30-Shear Forward Primer sequence’ provided in the ‘Intact Proviral DNA Assay (IPDA)’ section of the Methods, which incorrectly read ‘CCAATTTGCTGCTCCTTGGG’. The correct sequence of the ‘RPP30-Shear Forward Primer’ is ‘CCATTTGCTGCTCCTTGGG’. This has been corrected in both the PDF and HTML versions of the Article.

