# Supplementary material for: HIV-1 diversity considerations in the application of the Intact Proviral DNA Assay (IPDA)
Source: Nat Commun. 2021 Jan 8;12:165. doi: 10.1038/s41467-020-20442-3 (PMC7794580; doi:10.1038/s41467-020-20442-3)
Supplement: Supplementary file 1 — Supplementary Information [file 41467_2020_20442_MOESM1_ESM.pdf]

Supplementary Information

HIV Diversity Considerations in the Application of the Intact Proviral DNA Assay (IPDA)

Natalie N. Kinloch[1,2], Yanqin Ren[3], Winiffer D. Conce Alberto[3], Winnie Dong[2], Pragya Khadka[3], Szu Han Huang[3], Talia M. Mota[3], Andrew Wilson[4], Aniqa Shahid[1,2], Don Kirkby[2], Marianne Harris[2,5] Colin Kovacs[6], Erika Benko[6], Mario A. Ostrowski[7], Perla M. Del Rio Estrada[8], Avery Wimpelberg[9], Christopher Cannon[9], W. David Hardy[9], Lynsay MacLaren[9], Harris Goldstein[10], Chanson J. Brumme[2,5], Guinevere Q. Lee[3], Rebecca M. Lynch[4], Zabrina L. Brumme[1,2*], R. Brad Jones[3,4*]

[1] Faculty of Health Sciences, Simon Fraser University, Burnaby, Canada
[2] British Columbia Centre for Excellence in HIV/AIDS, Vancouver, Canada
[3] Infectious Diseases Division, Department of Medicine, Weill Cornell Medical College, New York, USA
[4] Department of Microbiology, Immunology and Tropical Medicine, George Washington University, Washington DC, USA
[5] Faculty of Medicine, University of British Columbia, Vancouver, Canada
[6] Maple Leaf Medical Clinic, Toronto, Canada
[7] Department of Medicine, University of Toronto, Toronto, Canada.
[8] Center for Research in Infectious Diseases, National Institute of Respiratory Diseases, Mexico City, Mexico
[9] Whitman Walker Health, Washington DC, USA.
[10] Department of Microbiology and Immunology, Albert Einstein College of Medicine, Bronx, NY, USA

These authors contributed equally: Natalie N. Kinloch, Yanqin Ren
[*] These authors jointly supervised this work: Zabrina L. Brumme, R. Brad Jones.
Correspondence and other requests should be addressed to: ZLB (zbrumme@sfu.ca) and/or RBJ (rbjones@med.cornell.edu)

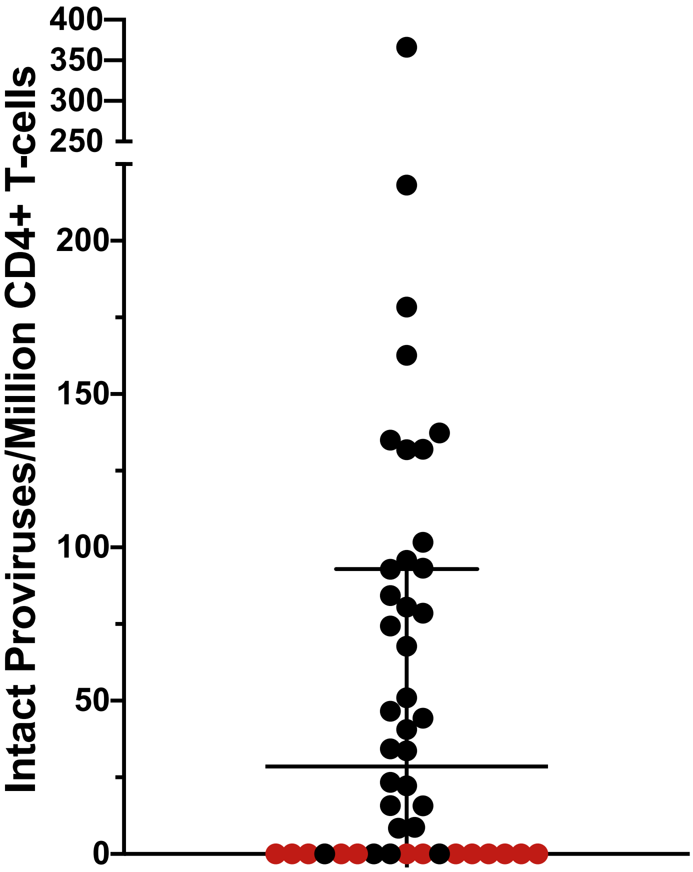

Supplementary Fig. 1: IPDA measurements from our North American cohort. Data derive from 46 unique study participants. Line and error bars indicate cohort median and interquartile range; red datapoints represent 13 presumed instances of IPDA detection failure. Data points show point estimate from 4 merged technical replicates for each participant sample. Source data are provided in the Source Data File.

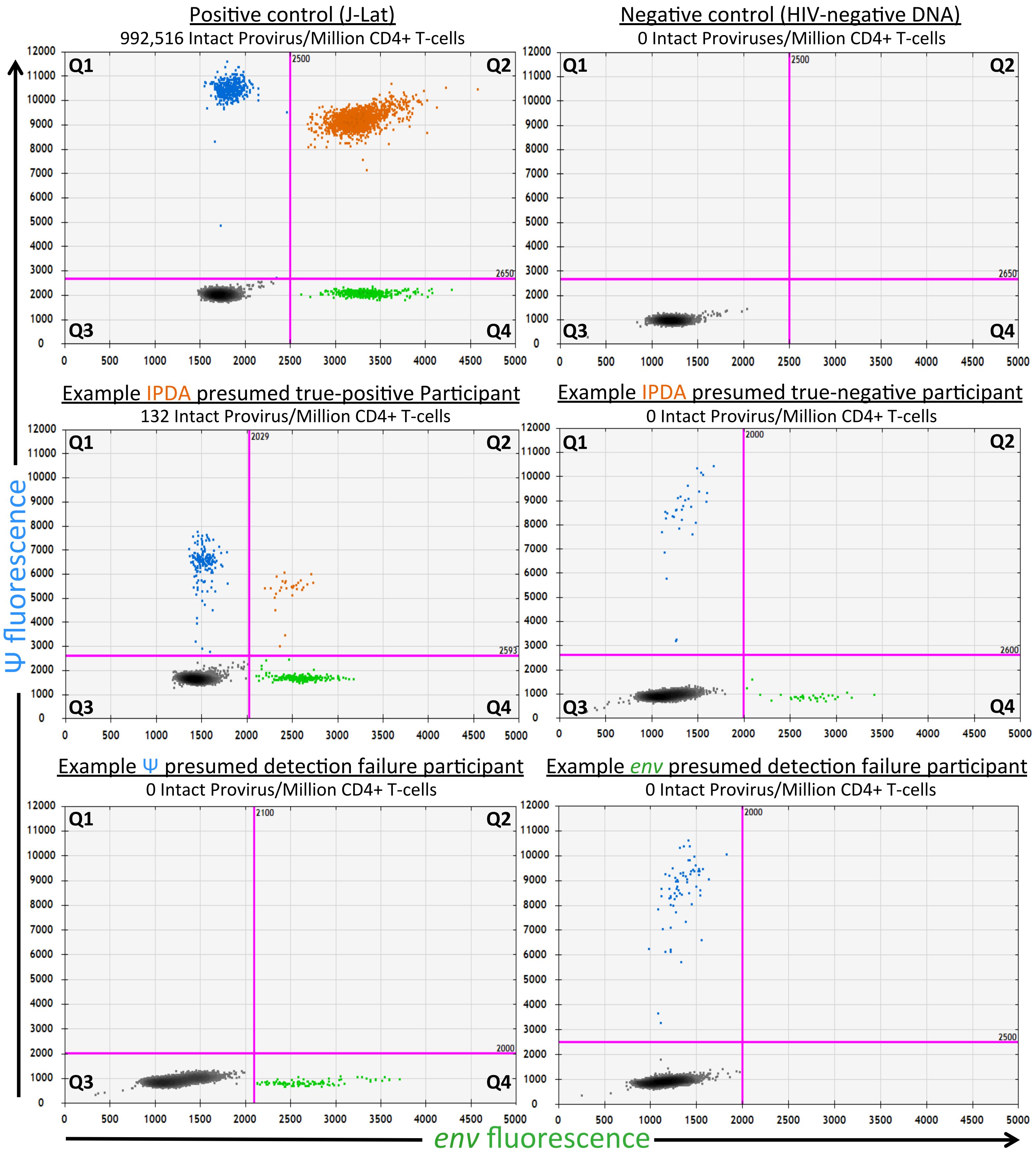

Supplementary Fig. 2: Example IPDA 2D plots for control and participant samples. 2D ddPCR plots showing Ψ-single positive events (Q1, blue), Ψ- and env- double positive events (Q2, orange), double-negative events (Q3, grey) and env-single positive events (Q4, green), for positive control J-Lat cell line (1 copy of HIV per cell, droplets in Q1 and Q4 are a result of anticipated DNA shearing that occurs during DNA extraction that is subsequently corrected mathematically based on RPP30 shearing) as well as samples from participants including a HIV-negative donor negative control; an IPDA true-positive participant; an IPDA true-negative participant; and two participants with IPDA detection failure. Plots show merge of 4 replicate wells.

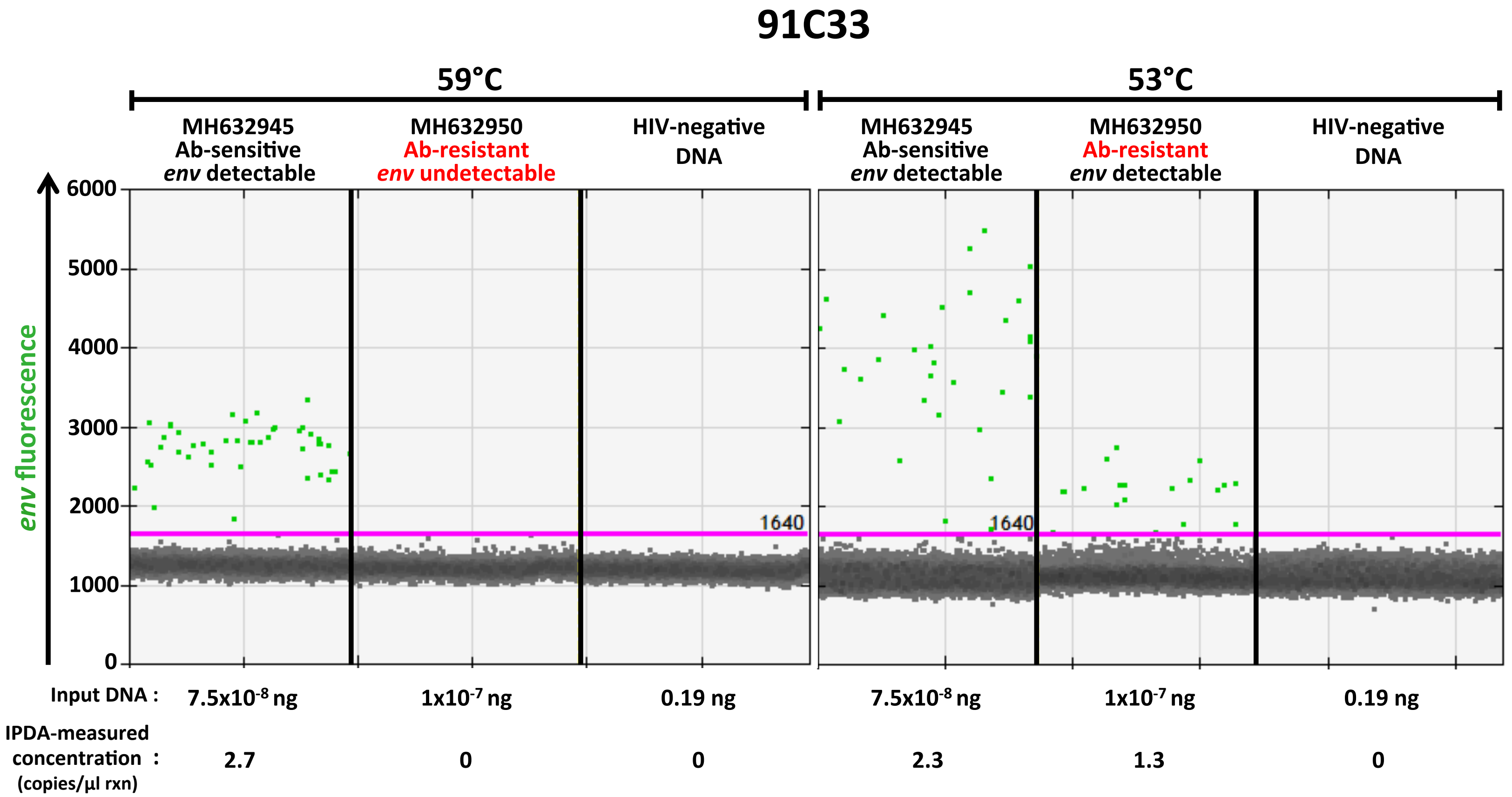

Supplementary Fig. 3: Modification of published IPDA conditions to reduce assay stringency allows detection of 91C33 mismatch variant. Representative ddPCR IPDA env plots from one of 2 independent experiments for MH632945 (representing 91C33's bNAb-sensitive, IPDA env probe-matching HIV subpopulation) and MH632950 (representing 91C33's bNAb-resistant, IPDA env probe-mismatched HIV subpopulation). Positive droplets are green and negative droplets are grey. Templates were purified env PCR products of equal length and comparable quantities. Results are shown under published conditions and when annealing/extension temperature was reduced to 53°C.

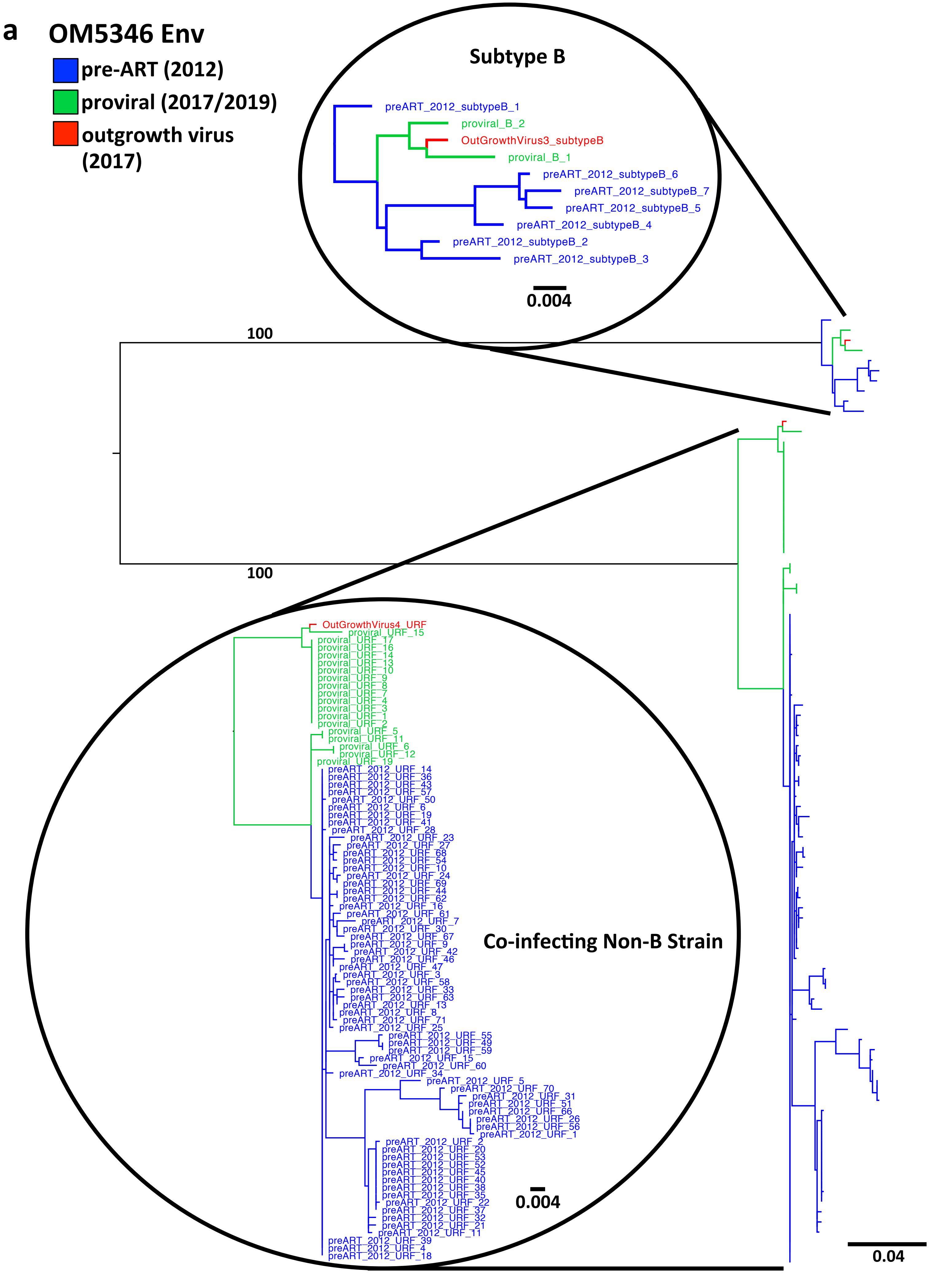
a
OM5346 Env
pre-ART (2012)
proviral (2017/2019)
outgrowth virus (2017)
Subtype B
preART_2012_subtypeB_1
proviral_B_2
OutGrowthVirus3_subtypeB
proviral_B_1
preART_2012_subtypeB_6
preART_2012_subtypeB_7
preART_2012_subtypeB_5
preART_2012_subtypeB_4
preART_2012_subtypeB_2
preART_2012_subtypeB_3
0.004
100
100
OutGrowthVirus4_URF
proviral_URF_15
proviral_URF_17
proviral_URF_16
proviral_URF_14
proviral_URF_13
proviral_URF_10
proviral_URF_9
proviral_URF_8
proviral_URF_7
proviral_URF_4
proviral_URF_3
proviral_URF_1
proviral_URF_2
proviral_URF_5
proviral_URF_11
proviral_URF_6
proviral_URF_12
proviral_URF_19
preART_2012_URF_14
preART_2012_URF_36
preART_2012_URF_43
preART_2012_URF_57
preART_2012_URF_50
preART_2012_URF_6
preART_2012_URF_19
preART_2012_URF_41
preART_2012_URF_28
preART_2012_URF_23
preART_2012_URF_27
preART_2012_URF_68
preART_2012_URF_54
preART_2012_URF_10
preART_2012_URF_24
preART_2012_URF_69
preART_2012_URF_44
preART_2012_URF_62
preART_2012_URF_16
preART_2012_URF_61
preART_2012_URF_7
preART_2012_URF_30
preART_2012_URF_67
preART_2012_URF_9
preART_2012_URF_42
preART_2012_URF_46
preART_2012_URF_47
preART_2012_URF_3
preART_2012_URF_58
preART_2012_URF_33
preART_2012_URF_63
preART_2012_URF_13
preART_2012_URF_8
preART_2012_URF_71
preART_2012_URF_25
preART_2012_URF_55
preART_2012_URF_49
preART_2012_URF_59
preART_2012_URF_15
preART_2012_URF_60
preART_2012_URF_34
preART_2012_URF_5
preART_2012_URF_70
preART_2012_URF_31
preART_2012_URF_51
preART_2012_URF_66
preART_2012_URF_26
preART_2012_URF_56
preART_2012_URF_1
preART_2012_URF_2
preART_2012_URF_20
preART_2012_URF_53
preART_2012_URF_52
preART_2012_URF_45
preART_2012_URF_40
preART_2012_URF_38
preART_2012_URF_35
preART_2012_URF_22
preART_2012_URF_37
preART_2012_URF_32
preART_2012_URF_21
preART_2012_URF_11
preART_2012_URF_39
preART_2012_URF_4
preART_2012_URF_18
Co-infecting Non-B Strain
0.004
0.04

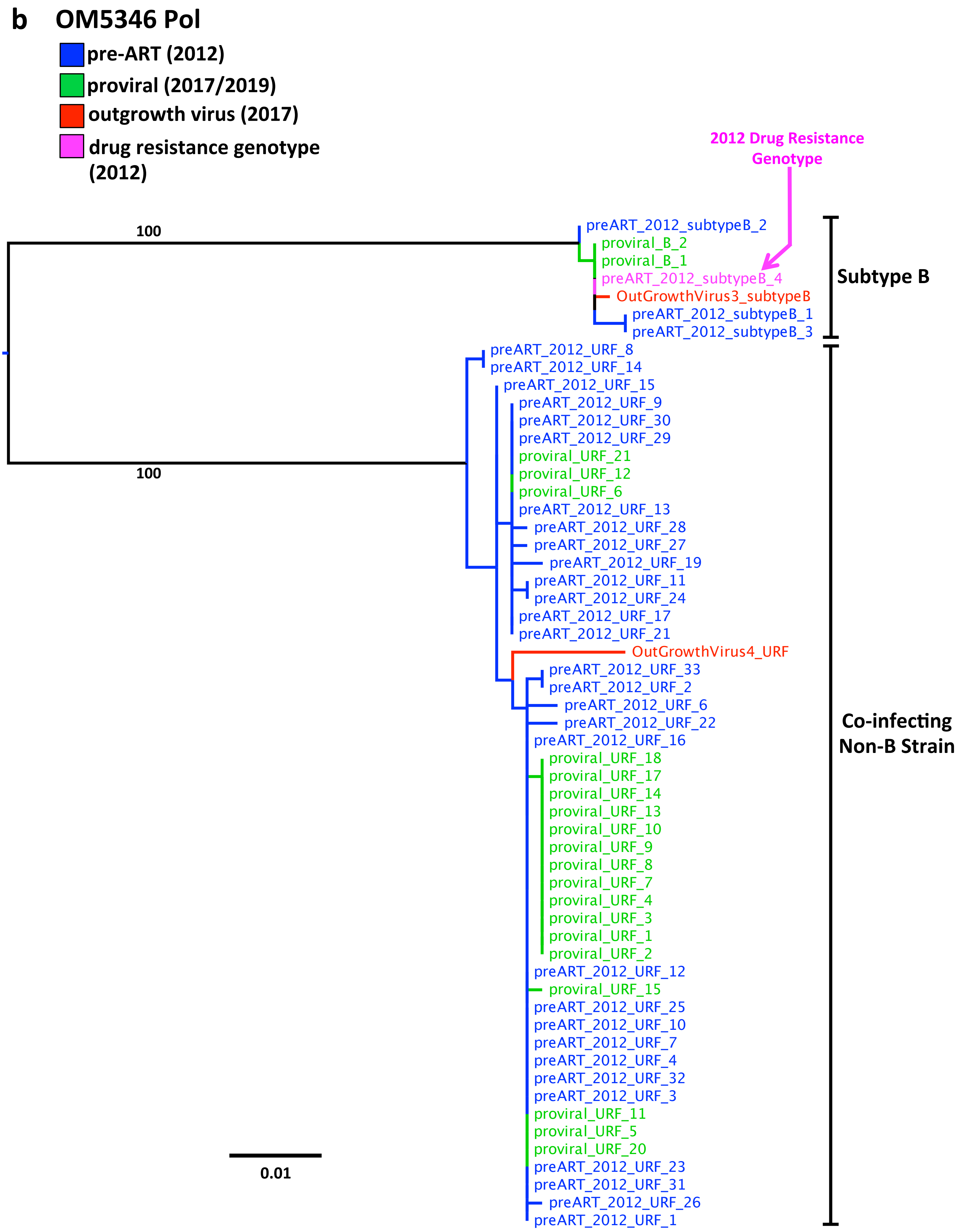

b OM5346 Pol
pre-ART (2012)
proviral (2017/2019)
outgrowth virus (2017)
drug resistance genotype (2012)
2012 Drug Resistance Genotype
100
100
preART_2012_subtypeB_2
proviral_B_2
proviral_B_1
preART_2012_subtypeB_4
OutGrowthVirus3_subtypeB
preART_2012_subtypeB_1
preART_2012_subtypeB_3
Subtype B
preART_2012_URF_8
preART_2012_URF_14
preART_2012_URF_15
preART_2012_URF_9
preART_2012_URF_30
preART_2012_URF_29
proviral_URF_21
proviral_URF_12
proviral_URF_6
preART_2012_URF_13
preART_2012_URF_28
preART_2012_URF_27
preART_2012_URF_19
preART_2012_URF_11
preART_2012_URF_24
preART_2012_URF_17
preART_2012_URF_21
OutGrowthVirus4_URF
preART_2012_URF_33
preART_2012_URF_2
preART_2012_URF_6
preART_2012_URF_22
preART_2012_URF_16
proviral_URF_18
proviral_URF_17
proviral_URF_14
proviral_URF_13
proviral_URF_10
proviral_URF_9
proviral_URF_8
proviral_URF_7
proviral_URF_4
proviral_URF_3
proviral_URF_1
proviral_URF_2
preART_2012_URF_12
proviral_URF_15
preART_2012_URF_25
preART_2012_URF_10
preART_2012_URF_7
preART_2012_URF_4
preART_2012_URF_32
preART_2012_URF_3
proviral_URF_11
proviral_URF_5
proviral_URF_20
preART_2012_URF_23
preART_2012_URF_31
preART_2012_URF_26
preART_2012_URF_1
Co-infecting Non-B Strain
0.01

Supplementary Fig. 4: Env and pol phylogenies for participant OM5346, who is co-infected with two HIV strains. **a** Maximum likelihood phylogeny inferred from single-genome-amplified env RNA sequences from pre-ART plasma (2012, blue), proviruses sampled on ART (2017 and 2019, green) and replication competent HIV sequences isolated from the reservoir (2017, red). Scale bars indicate substitutions per nucleotide site. Numbers on main branches indicate branch support values. **b** Same as a, but for HIV pol RNA sequences. This phylogeny additionally includes a sequence from a clinical HIV drug resistance test performed in 2012 (pink). Pol sequences of replication-competent reservoir sequences (2017, red) were recovered from cells infected with these viruses in vitro. Source data in the form of newick treefiles are provided in the Source Data File.

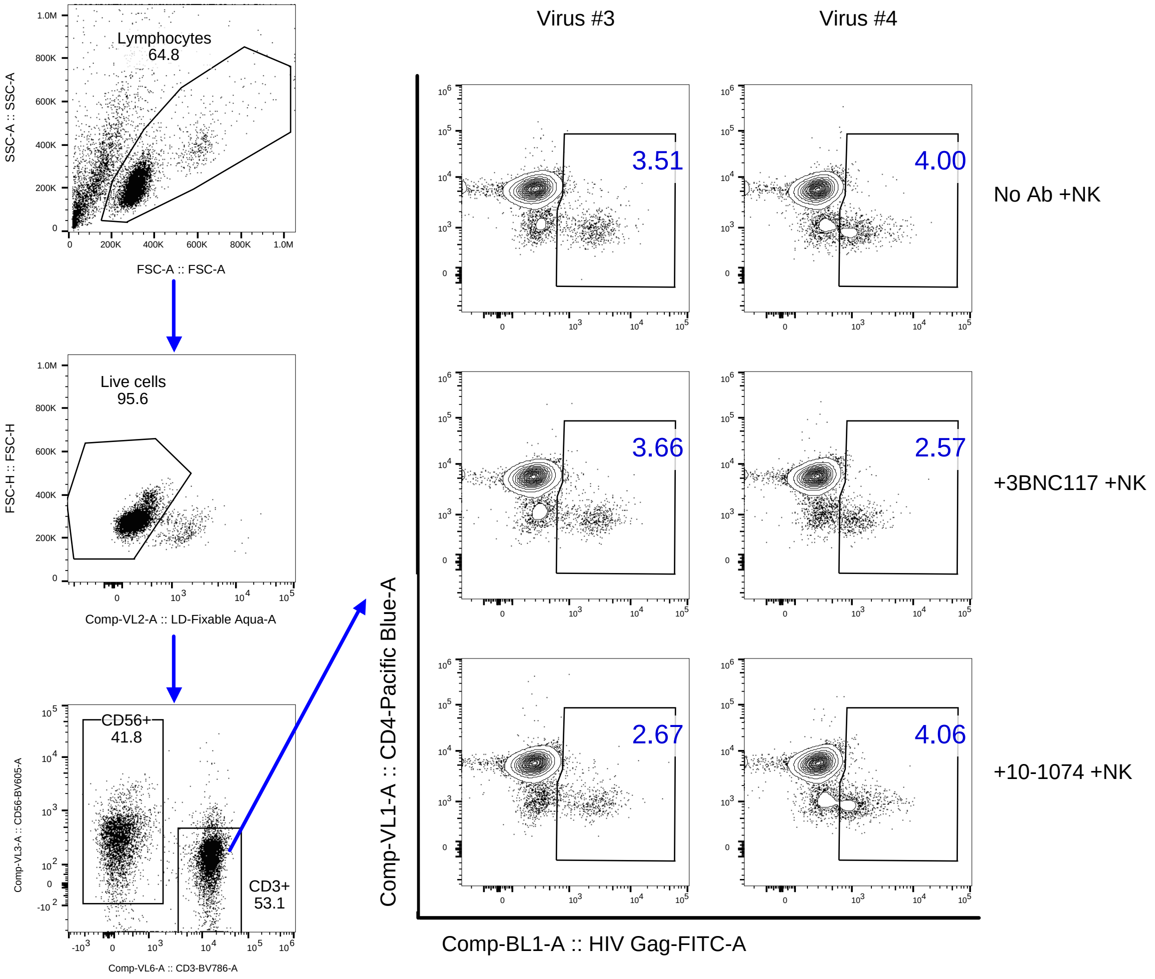

Supplementary Fig. 5: Gating strategy for the ADCC data shown in Fig 2c. Activated CD4+ T-cells from an HIV-negative donor were infected with OM5346 virus 3 or virus 4, and co-cultured with Natural Killer (NK) cells in the presence or absence of specific bNAbs to measure the percentage of infected cells remaining following ADCC-mediated killing. All samples were initially gated using forward and side scatter to identify lymphocytes, and then using viability dye to gate live cells. CD3 and CD56 were used to gate target cells (activated CD4+ T-cells; denoted by the CD3+ population in the figure) and effector cells (NK cells; denoted by the CD56+ population). From the CD3+ population, HIV-Gag and CD4 were used to identify the percentage of target cells that survived killing following infection with virus 3 or virus 4. Top row plots show CD4+ T cells remaining after addition of NK cells only (no antibody control), middle row plots show CD4+ T cells remaining after addition of NK cells and 3BNC117, bottom row plots show CD4+ T cells remaining after addition of NK cells and 3BNC117. Note that CD4 downregulation occurs in HIV-infected (HIV Gag+) cells due to CD4 downregulation induced by the viral accessory protein Nef. Plots show representative data from one of 2 independent experiments.

a Virus-infected cells: No detection of OM5346 Virus 4 by IPDA

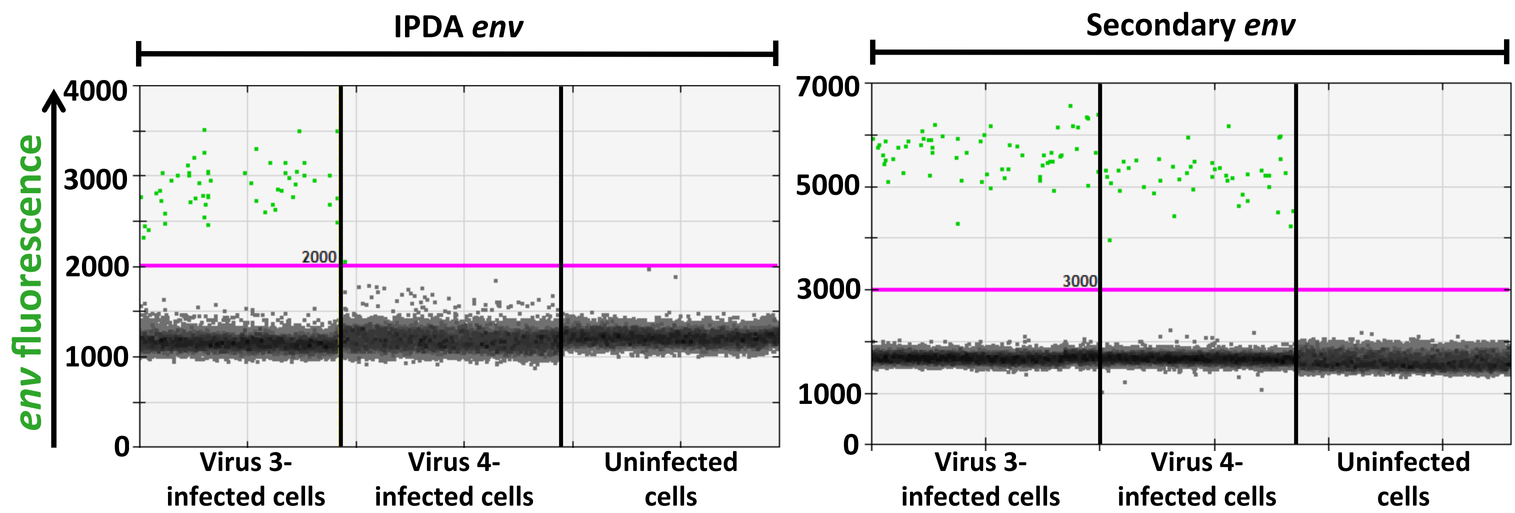

input DNA	0.75 ng	0.75 ng	0.75 ng		0.75 ng	0.75 ng	0.75 ng
IPDA-measured concentration (copies/ul rxn)	3.6	0.06	0		3.6	3.1	0

b Detection of Virus 4 pure template at high, but not biological, concentrations

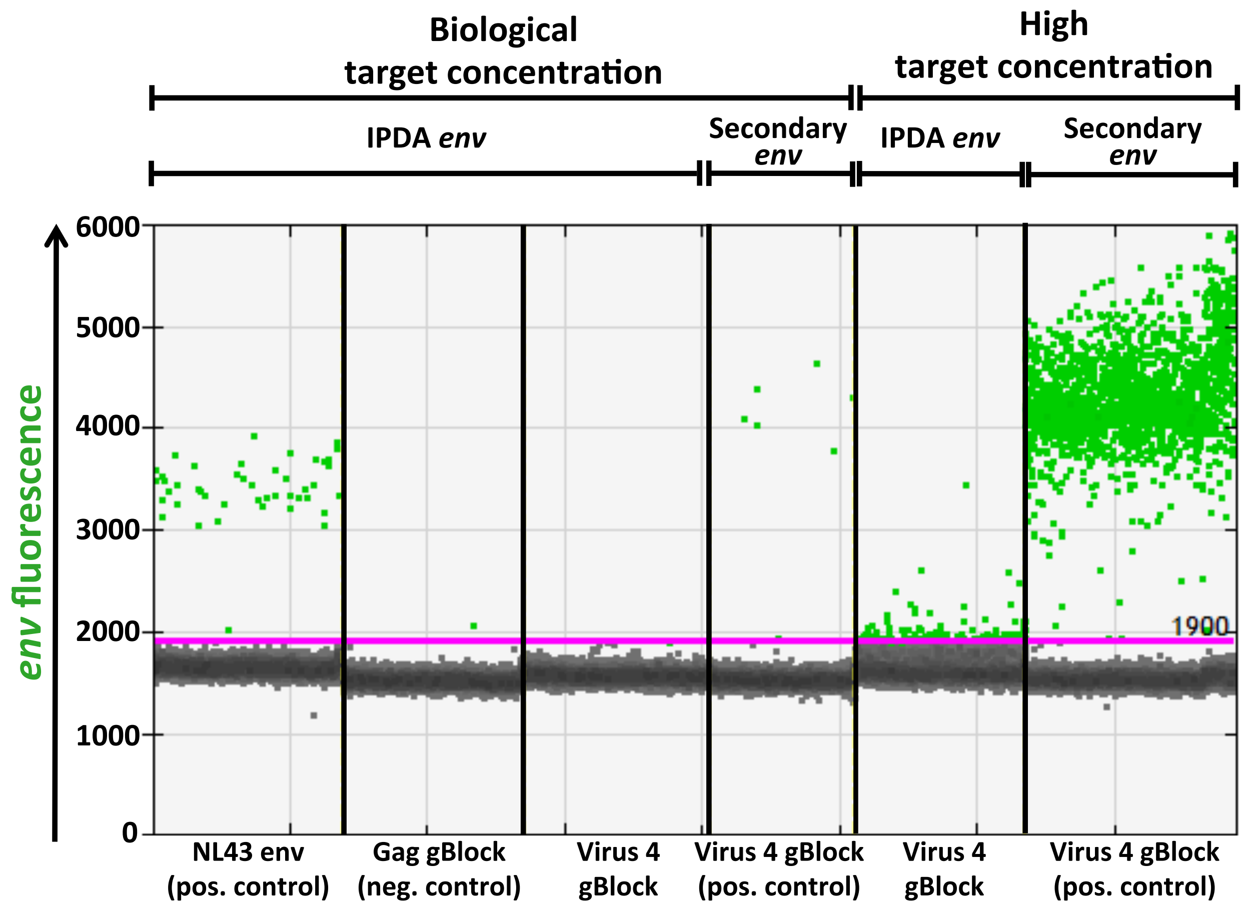

input DNA (nmol)	6.9×10^{-7}	6.9×10^{-7}	6.9×10^{-7}	6.9×10^{-7}	6.9×10^{-4}	6.9×10^{-4}
IPDA-measured concentration (copies/ul rxn)	3.8	0.09	0.09	0.8	15.1	162

Supplementary Fig. 6: OM5346 Virus 4 sequence is undetectable by the IPDA at biological concentrations. a. Representative 1D *env* plots using IPDA or the secondary *env* reaction from $CD4^+$ T-cells infected with OM5346 virus 3 (IPDA *env* probe match), virus 4 (IPDA *env* probe G13A mismatch) or uninfected cells (negative control). Positive droplets are green; negative droplets are grey. Copies/µl reaction as calculated from the experimental data are shown below the plots, which are from one of 2 independent experiments. The IPDA *env* data is the same as Figure 2d. **b.** Representative 1D *env* plots of OM5346 virus 4, tested as a synthetic DNA gene fragment (Virus 4 gBlock), using IPDA and secondary *env* reactions, at different input concentrations. A Gag synthetic gene fragment (Gag gBlock) served as the negative control. Input DNA quantity and copies/µl reaction as calculated from the experimental data are shown below the plots, which are from one of two technical replicates performed in a single experiment. These observations provide a possible explanation to reconcile the original authors' ability to detect this sequence using a pure plasmid template and our inability to detect it at concentrations mimicking a biological sample.

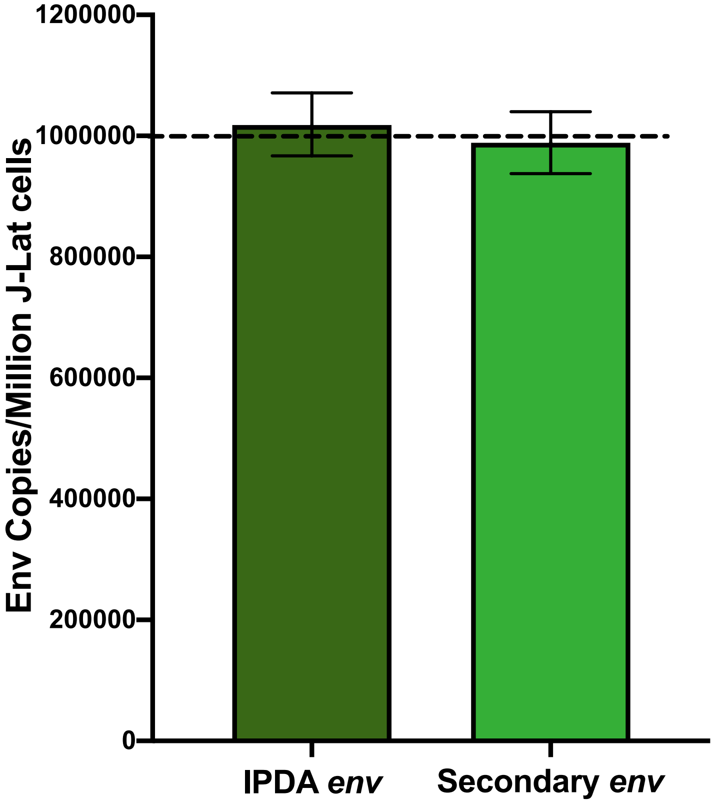

Supplementary Fig. 7: Comparable detection of expected 1:1 HIV-to-Cell ratio in J-Lat cells by IPDA and Secondary *env* reactions. Histogram height and error bars indicate the point estimate and 95% total Poisson confidence interval from 4 merged technical replicates from one of 3 independent experiments. Source data are provided in the Source Data File.
