# Supplementary material for: HIV-1 diversity considerations in the application of the Intact Proviral DNA Assay (IPDA)
Source: Nat Commun. 2021 Jan 8;12:165. doi: 10.1038/s41467-020-20442-3 (PMC7794580; doi:10.1038/s41467-020-20442-3)
Supplement: Supplementary file 2 — Reporting Summary [file 41467_2020_20442_MOESM2_ESM.pdf]

nature research

Corresponding author(s): Dr. R. Brad Jones/ Dr. Zabrina Brumme

Last updated by author(s): Nov 20, 2020

Reporting Summary

Nature Research wishes to improve the reproducibility of the work that we publish. This form provides structure for consistency and transparency in reporting. For further information on Nature Research policies, see Authors & Referees and the Editorial Policy Checklist .

Statistics

For all statistical analyses, confirm that the following items are present in the figure legend, table legend, main text, or Methods section.

n/a	Confirmed	
☐	☒	The exact sample size (n) for each experimental group/condition, given as a discrete number and unit of measurement
☐	☒	A statement on whether measurements were taken from distinct samples or whether the same sample was measured repeatedly
☐	☒	The statistical test(s) used AND whether they are one- or two-sided<br>*Only common tests should be described solely by name; describe more complex techniques in the Methods section.*
☒	☐	A description of all covariates tested
☒	☐	A description of any assumptions or corrections, such as tests of normality and adjustment for multiple comparisons
☐	☒	A full description of the statistical parameters including central tendency (e.g. means) or other basic estimates (e.g. regression coefficient) AND variation (e.g. standard deviation) or associated estimates of uncertainty (e.g. confidence intervals)
☐	☒	For null hypothesis testing, the test statistic (e.g. F, t, r) with confidence intervals, effect sizes, degrees of freedom and P value noted<br>*Give P values as exact values whenever suitable.*
☒	☐	For Bayesian analysis, information on the choice of priors and Markov chain Monte Carlo settings
☒	☐	For hierarchical and complex designs, identification of the appropriate level for tests and full reporting of outcomes
☐	☒	Estimates of effect sizes (e.g. Cohen's d, Pearson's r), indicating how they were calculated

Our web collection on statistics for biologists contains articles on many of the points above.

Software and code

Policy information about availability of computer code

Data collection	ddPCR data was collected using QuantaSoft (BioRad Laboratories, Inc., version 1.7.4). Flow cytometry data was collected using Attune NxT Software (ThermoFisher Scientific, version 2.7)
Data analysis	ddPCR data was analyzed using QuantaSoft (BioRad Laboratories, Inc., version 1.7.4). Infectious Units/Million CD4+ T-cells (QVOA readout) was determined using the web platform of the Extreme Limiting Dilution Analysis (ELDA) software hosted by the Walter and Eliza Hall Institute for Medical Research (version 1.0, PMID: 19567251). Flow cytometry data was analyzed using FlowJo (Becton Dickinson, version 10.6). Sanger sequence data was analyzed using Sequencher (GeneCodes, version 5.0.1). Illumina MiSeq data was de novo assembled using the custom software MiCall, available at https://github.com/cfe-lab/MiCall, which features an in-house modification of the Iterative Virus Assembler (IVA) (PMID: 25725497), or through collaboration with the Massachusetts General Hospital CCIB Core. HIV pol and env regions were excised using GeneCutter (version 1, https://www.hiv.lanl.gov/content/sequence/GENE_CUTTER/cutter.html). Nucleotide sequences were multiply-aligned using MAFFT (version 7.427, PMID: 233296090) and inspected using AliView (version 1.19, PMID: 25095880). Maximum-likelihood phylogenies were inferred using PhyML (version 3.0, PMID:20525638) and visualized in FigTree (version 1.3.1). All statistical analyses were performed using GraphPad Prism (version 8).

For manuscripts utilizing custom algorithms or software that are central to the research but not yet described in published literature, software must be made available to editors/reviewers. We strongly encourage code deposition in a community repository (e.g. GitHub). See the Nature Research guidelines for submitting code & software for further information.

Data

Policy information about availability of data

All manuscripts must include a data availability statement. This statement should provide the following information, where applicable:
- Accession codes, unique identifiers, or web links for publicly available datasets
- A list of figures that have associated raw data
- A description of any restrictions on data availability

All data that support the findings of this study are available in the manuscript, Figures and Supplementary Figures. Source data for Figs. 1b, 1d, 1e, 2c and 4 and

Supplementary Figs. 1, 4 and 7 are also provided with this paper. HIV sequences used for phylogenetic inference are available in GenBank under accession numbers MT792083-MT792233; HIV sequences for participant 91C33 were previously published under GenBank accession numbers MH632930- MH63295515. HIV-1 subtype B sequences used to identify the location for the secondary env primer/probe set are available through the Los Alamos National Laboratory HIV Sequence database (https://www.hiv.lanl.gov/components/sequence/HIV/search/search.html). Additional information is available from the corresponding authors upon request.

Field-specific reporting

Please select the one below that is the best fit for your research. If you are not sure, read the appropriate sections before making your selection.

☒ Life sciences ☐ Behavioural & social sciences ☐ Ecological, evolutionary & environmental sciences

For a reference copy of the document with all sections, see nature.com/documents/nr-reporting-summary-flat.pdf

Life sciences study design

All studies must disclose on these points even when the disclosure is negative.

Sample size	N=46 virally-suppressed individuals with HIV were included in the present study testing the Intact Proviral DNA Assay (IPDA). The Quantitative Viral Outgrowth Assay was performed on a subset thereof (N=37), where sufficient biological material was available. This sample size was chosen as it is comparable to that of the original description of the IPDA (n= 62, Bruner et al., PMID: 30700913) and subsequent follow up studies. This sample size further reflected the number of participants in our cohorts with sufficient biological material available to perform the IPDA
Data exclusions	No data were excluded from this study
Replication	Four technical replicates of the IPDA were performed for each participant. A minimum of 4 technical replicates of the HIV Gag ddPCR assay were performed for each participant. A minimum of 12 technical replicates at each dilution in the QVOA assay were performed. Two independent experiments were performed for the ADCC assay, where representative data from three technical replicates of one experiment are shown. Three independent experiments comparing the performance of the IPDA and secondary env primer/probe sets were performed, each with 4 technical replicates. Results of all replicate experiments were consistent with each other and successful.
Randomization	Not applicable. Division of participants/samples into different conditions was not necessary to address the research question in this study.
Blinding	Not applicable as participants/samples were not divided into different conditions in this study

Reporting for specific materials, systems and methods

We require information from authors about some types of materials, experimental systems and methods used in many studies. Here, indicate whether each material, system or method listed is relevant to your study. If you are not sure if a list item applies to your research, read the appropriate section before selecting a response.

Materials & experimental systems

n/a	Involved in the study	
☐	☒	Antibodies
☐	☒	Eukaryotic cell lines
☒	☐	Palaeontology
☒	☐	Animals and other organisms
☐	☒	Human research participants
☒	☐	Clinical data

Methods

n/a	Involved in the study	
☒	☐	ChIP-seq
☐	☒	Flow cytometry
☒	☐	MRI-based neuroimaging

Antibodies

Antibodies used	For cell activation: anti-human CD3 (Biolegend, catalogue number: 317326, clone: OKT3), CD28 (Biolegend, catalogue number: 302934, clone: CD28.2)<br>For ADCC experiments: 3BNC117 (NIH AIDS reagent program, catalogue number: 12474), 10-1074 (NIH AIDS reagent program, catalogue number: 12477)<br>For flow cytometry: anti-human IgG (Southern BioTech, catalogue number: 2040-31), CD3 (Biolegend, catalogue number: 344842, clone SK7), CD56 (Biolegend, catalogue number: 318334, clone HCD56), CD4 (Biolegend, catalogue number 200521, clone: RPA-T4), HIV p24 (Beckman Coulter, catalogue number: 6604665, clone KC57)
Validation	For cell activation:<br><br>anti-human CD3 (Biolegend, catalogue number: 317326, clone: OKT3)<br>The OKT3 monoclonal antibody reacts with an epitope on the epsilon-subunit within the human CD3 complex. Clone OKT3 can

block the binding of clones SK7 and UCHT1.4 The OKT3 antibody is able to induce T cell activation. Additional reported applications (for the relevant formats) include: immunohistochemical staining of acetone-fixed frozen sections and activation of T cells.

Application References:

1. Schlossman S, et al. Eds. 1995. Leucocyte Typing V. Oxford University Press. New York.
2. Knapp W. 1989. Leucocyte Typing IV. Oxford University Press New York.
3. Barclay N, et al. 1997. The Leucocyte Antigen Facts Book. Academic Press Inc. San Diego.
4. Li B, et al. 2005. Immunology 116:487.
5. Jeong HY, et al. 2008. J. Leuckocyte Biol. 83:755. PubMed
6. Alter G, et al. 2008. J. Virol. 82:9668. PubMed
7. Manevich-Mendelson E, et al. 2009. Blood 114:2344. PubMed
8. Pinto JP, et al. 2010. Immunology. 130:217. PubMed
9. Biggs MJ, et al. 2011. J. R. Soc. Interface. 8:1462. PubMed

anti-human CD28 (Biolegend, catalogue number: 302934, clone: CD28.2)

Each lot of this antibody is quality control tested by immunofluorescent staining with flow cytometric analysis. Additional reported applications (for the relevant formats) include: immunoprecipitation, immunohistochemical staining of acetone-fixed frozen tissue sections4, and in vitro T cell costimulation5-8. The CD28.2 antibody co-stimulates T cell proliferation and cytokine production in the presence of suboptimal amounts of anti-CD3 antibody. For highly sensitive assays, we recommend Ultra-LEAF™ purified antibody (Cat. No. 302934) with a lower endotoxin limit than standard LEAF™ purified antibodies (Endotoxin <0.01 EU/µg).

Application References:

1. Schlossman S, et al. Eds. 1995. Leucocyte Typing V. Oxford University Press. New York.
2. Nunes J, et al. 1993. Biochem. J. 293:835.
3. Calea-Lauri J, et al. 1999. J. Immunol. 163:62.
4. Tazi A, et al. 1999. J. Immunol. 163:3511. (IHC)
5. Marti F, et al. 2001. J. Immunol. 166:197. (Costim)
6. Jeong SH, et al. 2004. J. Virol. 78:6995. (Costim)
7. Rivollier A, et al. 2004. Blood 104:4029. (Costim)
8. Scharschmidt E, et al. 2004. Mol. Cell Biol. 24:3860. (Costim)
9. Sheng W, et al. 2007.Elsevier 580:6819. PubMed
10. Mitsuhashi M. 2007. Clin Chem.53:148. PubMed
11. Ye Z, et al. 2008. Infect. Immun. 76:2541. PubMed
12. Magatti M, et al. 2008. Stem Cells 26:182. (FA) PubMed

For ADCC experiments:

3BNC117 (NIH AIDS reagent program, catalogue number: 12474)

This recombinant antibody was produced in a FreeStyle 239 expression system and purified using MEP capture and Protein G affinity purification. This antibody originates from an HIV-1 infected male from the United States. This antibody potently neutralizes a broad variety of laboratory HIV-1 strains and primary isolates.

References:

Shingai, M., Nishimura, Y., Klein, F., Mouquet, H., Donau, O. K., Plishka, R., . . . Martin, M. A. (2013). Antibody-mediated immunotherapy of macaques chronically infected with SHIV suppresses viraemia. Nature, 503(7475), 277-280. doi:10.1038/nature12746 PUBMED

Scheid, J. F., Mouquet, H., Ueberheide, B., Diskin, R., Klein, F., Oliveira, T. Y., . . . Nussenzweig, M. C. (2011). Sequence and structural convergence of broad and potent HIV antibodies that mimic CD4 binding. Science, 333(6049), 1633-1637. doi:10.1126/science.1207227 PUBMED

10-1074 (NIH AIDS reagent program, catalogue number: 12477)

This recombinant antibody was produced in an Expi293F expression system and was two-step purified by MabSelect SuReTM LX and HiLoad26/600. This antibody originates from an HIV-1 infected individual. This antibody has both potent and broad neutralization.

References:

Shingai, M., Nishimura, Y., Klein, F., Mouquet, H., Donau, O. K., Plishka, R., Buckler-White, A., Seaman, M., Piatak, M., Jr., Lifson, J. D., Dimitrov, D. S., Nussenzweig, M. C. and Martin, M. A. (2013). Antibody-mediated immunotherapy of macaques chronically infected with SHIV suppresses viraemia. Nature, 503(7475), 277-80. doi:10.1038/nature12746 PUBMED

Mouquet, H., Scharf, L., Euler, Z., Liu, Y., Eden, C., Scheid, J. F., Halper-Stromberg, A., Gnanapragasam, P. N., Spencer, D. I., Seaman, M. S., Schuitemaker, H., Feizi, T., Nussenzweig, M. C. and Bjorkman, P. J. (2012). Complex-type N-glycan recognition by potent broadly neutralizing HIV antibodies. Proc Natl Acad Sci U S A, 109(47), E3268-77. doi:10.1073/pnas.1217207109 PUBMED

For flow cytometry:

anti-human IgG (Southern BioTech, catalogue number: 2040-31)

Validated by manufacturer based on binding to an ELISA plate coated with purified human IgG, IgM, and IgA. Quality tested applications for relevant formats include -ELISA 2-5, FLISA 6, Flow Cytometry 1,10,13,14. Other referenced applications for

relevant formats include -ELISpot 2,3,11, Immunohistochemistry-Frozen Sections 7,8, Immunohistochemistry-Paraffin Sections 9, Immunocytochemistry 10-12, Western Blot 4,12,17,18, Immunoprecipitation 19, Multiplex 3,15,16, Depletion 14,20

References

1. Gilewski T, Adluri S, Ragupathi G, Zhang S, Yao T, Panageas K, et al. Vaccination of high-risk breast cancer patients with mucin-1 (MUC1) keyhole limpet hemocyanin conjugate plus QS-21. Clin Cancer Res. 2000;6:1693-1701. (FC)

2. Traggiai E, Volpi S, Schena F, Gattorno M, Ferlito F, Moretta L, et al. Bone marrow-derived mesenchymal stem cells induce both polyclonal expansion and differentiation of B cells isolated from healthy donors and systemic lupus erythematosus patients. Stem Cells. 2008;26:562-9. (ELISA, ELISPOT)

3. Staats HF, Kirwan SM, Whisnant CC, Stephenson JL, Wagener DK, Majumder PP. Development of a bead immunoassay to measure Vi polysaccharide-specific serum IgG after vaccination with the Salmonella enterica serovar Typhi Vi polysaccharide. Clin Vaccine Immunol. 2010;17:412-9. (ELISA, Multiplex)

4. Lai H, Engle M, Fuchs A, Keller T, Johnson S, Gorlatov S, et al. Monoclonal antibody produced in plants efficiently treats West Nile virus infection in mice. Proc Natl Acad Sci USA. 2010;107:2419-24. (ELISA, WB)

5. Kwa S, Lai L, Gangadhara S, Siddiqui M, Pillai VB, Labranche C, et al. CD40L-adjuvanted DNA/modified vaccinia virus Ankara simian immunodeficiency virus SIV239 vaccine enhances SIV-specific humoral and cellular immunity and improves protection against a heterologous SIVE660 mucosal challenge. J Virol. 2014;88:9579-89. (ELISA)

6. Spensieri F, Borgogni E, Zedda L, Bardelli M, Buricchi F, Volpini G, et al. Human circulating influenza-CD4+ ICOS1+IL-21+ T cells expand after vaccination, exert helper function, and predict antibody responses. Proc Natl Acad Sci USA. 2013;110:14330-5. (FLISA)

7. Borrego L, Maynard B, Peterson EA, George T, Iglesias L, Peters MS, et al. Deposition of eosinophil granule proteins precedes blister formation in bullous pemphigoid. Comparison with neutrophil and mast cell granule proteins. Am J Pathol. 1996;148:897-909. (IHC-FS)

8. Forshammar J, Isaksson S, Strid H, Stotzer P, Sjövall H, Simrén M, et al. A pilot study of colonic B cell pattern in irritable bowel syndrome. Scand J Gastroenterol. 2008;43:1461-6. (IHC-FS)

9. Rowley AH, Shulman ST, Mask CA, Finn LS, Terai M, Baker SC, et al. IgA plasma cell infiltration of proximal respiratory tract, pancreas, kidney, and coronary artery in acute Kawasaki disease. J Infect Dis. 2000;182:1183-91. (IHC-PS)

10. Oritani K, Kincade PW. Identification of stromal cell products that interact with pre-B cells. J Cell Biol. 1996;134:771-82. (ICC, FC)

11. Alwayn IP, Xu Y, Basker M, Wu C, Buhler L, Lambrigts D, et al. Effects of specific anti-B and/or anti-plasma cell immunotherapy on antibody production in baboons: depletion of CD20- and CD22-positive B cells does not result in significantly decreased production of anti-αGal antibody. Xenotransplantation. 2001;8:157-71. (ICC, ELISPOT)

12. Hasegawa H, Forte C, Barber I, Turnbaugh S, Stoops J, Shen M, et al. Modulation of in vivo IgG crystallization in the secretory pathway by heavy chain isotype class switching and N-linked glycosylation. Biochim Biophys Acta. 2014;1843:1325-38. (ICC, WB)

13. Kudo K, Imai C, Lorenzini P, Kamiya T, Kono K, Davidoff AM, et al. T lymphocytes expressing a CD16 signaling receptor exert antibody-dependent cancer cell killing. Cancer Res. 2014;74:93-103. (FC)

14. Dryer RL, Covey LR. Use of chromatin immunoprecipitation (ChIP) to detect transcription factor binding to highly homologous promoters in chromatin isolated from unstimulated and activated primary human B cells. Biol Proced Online. 2006;8:44-54. (FC, Depletion)

15. Pochechueva T, Chinarev A, Bovin N, Fedier A, Jacob F, Heinzelmann-Schwarz V. PEGylation of microbead surfaces reduces unspecific antibody binding in glycan-based suspension array. J Immunol Methods. 2014;412:42-52. (Multiplex)

16. Pickering JW, Larson MT, Martins TB, Copple SS, Hill HR. Elimination of false-positive results in a luminex assay for pneumococcal antibodies. Clin Vaccine Immunol. 2010;17:185-9. (Multiplex)

17. Tran M, Zhou B, Pettersson PL, Gonzalez MJ, Mayfield SP. Synthesis and assembly of a full-length human monoclonal antibody in algal chloroplasts. Biotechnol Bioeng. 2009;104:663-73. (WB)

18. Phoolcharoen W, Bhoo SH, Lai H, Ma J, Arntzen CJ, Chen Q, et al. Expression of an immunogenic Ebola immune complex in Nicotiana benthamiana. Plant Biotechnol J. 2011;9:807-16. (WB)

19. Santiago T, Kulemzin SV, Reshetnikova ES, Chikaev NA, Volkova OY, Mechetina LV, et al. FCRLA is a resident endoplasmic reticulum protein that associates with intracellular Igs, IgM, IgG and IgA. Int Immunol. 2011;23:43-53. (IP)

20. Palaia JM, McConnell M, Achenbach JE, Gustafson CE, Stoermer KA, Nolan M, et al. Neutralization of HIV subtypes A and D by breast milk IgG from women with HIV infection in Uganda. J Infect. 2014;68:264-72. (Depletion)

anti-human CD3 (Biolegend, catalogue number: 344842, clone SK7)

Each lot of this antibody is quality control tested by immunofluorescent staining with flow cytometric analysis

References:

1. Kan EA, et al. 1983. J. Immunol. 131:536.

2. Wood GS, et al. 1985. Am. J. Pathol. 120:371.

3. Van Dongen JJM, et al. 1988. Blood 71:603. (WB)

4. Haringman JJ, et al. 2005. Arthritis Res. Ther. 7:R862. (IHC)

5. Carbone A, et al. 1999. Blood 93:2319. (IHC)

6. Goval JJ, et al. 2006. J. Histochem. Cytochem. 54:75. (IF)

7. Rutjens E, et al. 2007. J. Immunol. 178:1702.

8. Kap Y, et al. 2009. J. Histochem. Cytochem. 57:1159. (IHC)

9. Yoshino N, et al. 2000. Exp. Anim. (Tokyo) 49:97. (FC)

anti-human CD56 (Biolegend, catalogue number: 318334, clone HCD56)

Each lot of this antibody is quality control tested by immunofluorescent staining with flow cytometric analysis.

References:

1. Bigley A, et al. 2013. Brain Behav Immun. 889:1591. PubMed

2. Du J, et al. 2014. Cancer Immunol Res. 2:878. PubMed

3. Jansen D, et al. 2015. Rheumatology. 54:728. PubMed
4. Grove K, et al. 2016. PLoS One. 11: 0145961. PubMed
5. Grenga I, et al. 2016. Clin Transl Immunology. 0.265972222. PubMed
6. Kritikou J, et al. 2016. Sci Rep. 6:30636. PubMed
7. Martrus G, et al. 2017. PLoS One.. 10.1371/journal.pone.0182532. PubMed
8. Jackson E, et al. 2017. PLoS One. 10.1371/journal.pone.0185160. PubMed
9. Garfall AL, et al. 2018. JCI Insight. 3. PubMed
10. Rydbirk R, et al. 2019. Sci Rep. 9:7781. PubMed
11. Fu J et al. 2019. Cell stem cell. 24(2):227-239 . PubMed

anti-human CD4 (Biolegend, catalogue number 200521, clone: RPA-T4)
Each lot of this antibody is quality control tested by immunofluorescent staining with flow cytometric analysis.
References:
1. Knapp W, et al. 1989. Leucocyte Typing IV. Oxford University Press. New York. (Activ)
2. Moir S, et al. 1999. J. Virol. 73:7972. (Activ)
3. Deng MC, et al. 1995. Circulation 91:1647. (IHC)
4. Friedman T, et al. 1999. J. Immunol. 162:5256. (IHC)
5. Mack CL, et al. 2004. Pediatr. Res. 56:79. (IHC)
6. Lan RY, et al. 2006. Hepatology 43:729.
7. Zenaro E, et al. 2009. J. Leukoc. Biol. 86:1393. (FC) PubMed
8. Yoshino N, et al. 2000. Exp. Anim. (Tokyo) 49:97. (FC)
9. Stoeckius M, et al. 2017. Nat. Methods. 14:865. (PG)

anti-HIV p24 (Beckman Coulter, catalogue number: 6604665, clone KC57)
Validated by the manufacturer based on positive staining of the HIV-infected 8E5 cell line.
References:
1. Jones RB, et al, 2016. A Subset of Latency-Reversing Agents Expose HIV-Infected Resting CD4+ T-Cells to Recognition by Cytotoxic T-Lymphocytes. PLoS Pathogens https://doi.org/10.1371/journal.ppat.1005545

Eukaryotic cell lines

Policy information about cell lines

Cell line source(s)	J-lat 9.2 cell line: obtained from the NIH AIDS Reagent Program (catalogue number: 9848), Division of AIDS, NIAID, NIH<br>MOLT-4 CCR5 cell line: obtained from the NIH AIDS Reagent Program (catalogue number: 4984), NIAID, NIH
Authentication	J- Lat 9.2 cell line: The expected HIV:Cell 1:1 ratio was observed in ddPCR experiments performed in this manuscript (Supplementary Fig. 7). Near-full HIV genome amplification and sequencing confirmed presence of an integrated HIV provirus in the cell line harboring green fluorescent protein (GFP) in place of the HIV Nef gene.<br>MOLT-4/CCR5 cell-line: CCR5 staining confirmed expression of this cell surface receptor. HLA Class I B sequence-based typing was performed and HLA type match that published for the cell line (B*57:01/B*18:01).
Mycoplasma contamination	J-Lat 9.2 cell line: None, confirmed using the Mycoplasma PCR Detection Kit (ABM, Cat #: G238)<br>MOLT-4 CCR5 cell line: None, confirmed using the Mycoplasma PCR Detection Kit (ABM, Cat #: G238)
Commonly misidentified lines<br>(See ICLAC register)	none

Human research participants

Policy information about studies involving human research participants

Population characteristics	Our cohort consisted of n= 46 virally-suppressed individuals with HIV (n=45 anti-retroviral [ARV]-suppressed, n=1 elite controller). Individuals were a median 54 (interquartile range [IQR]: 44-57) years old and 96% of participants were male. In ARV-suppressed participants, duration of untreated infection ranged from less than 6 months to more than 10 years. All participants were virally -suppressed for greater than 6 months at time of sampling, where individuals on ARVs where suppressed for a median of 9 (IQR: 5- 12) years at time of sampling. The median (IQR) nadir CD4+ T-cell count of study participants was 270 (105-375) cells/mm3, while the median (IQR) proximal CD4+ T-cell count was 674 (514- 863) cells/mm3.
Recruitment	Participants were recruited to cohorts in Vancouver, Toronto, New York, Washington and Mexico City by self-identification after viewing study recruitment materials in HIV care clinics that outlined the study purpose and major eligibility criteria (persons living with HIV with long-term suppressed viremia) and invited interested persons to contact the study team for more information. Recruited participants broadly reflect the demographics at each recruitment site/country, but otherwise we do not anticipate any overt biases specifically related to recruitment that could impact study results.
Ethics oversight	Ethics approval to conduct this study was obtained from the Institutional Review Boards of Simon Fraser University, Providence Health Care/ University of British Columbia, Weill Cornell Medicine, and the George Washington University. All participants provided written informed consent.

Note that full information on the approval of the study protocol must also be provided in the manuscript.

Flow Cytometry

Plots

Confirm that:

- [x] The axis labels state the marker and fluorochrome used (e.g. CD4-FITC).
- [x] The axis scales are clearly visible. Include numbers along axes only for bottom left plot of group (a 'group' is an analysis of identical markers).
- [x] All plots are contour plots with outliers or pseudocolor plots.
- [x] A numerical value for number of cells or percentage (with statistics) is provided.

Methodology

Sample preparation	HIV-negative CD4+ T cells were enriched from Peripheral Blood Mononuclear Cells (PBMCs) and activated with anti-CD3/CD28 antibodies for 48 hours, then infected with reactivated OM5346 viruses (#3 & #4) collected from QVOA supernatant. When the infection rate went up to over 5%, broadly neutralizing antibody (3BNC117 or 10-1074) or negative control medium solution was added and incubated for 2 hours, following which purified NK cells were added and incubated for 16 hours. Cells were stained with fluorophore-conjugated antibodies against human IgG, CD3, CD56, and CD4, as well as intracellular Gag and LIVE/DEAD Fixable Aqua Stain amine-reactive dye.
Instrument	Attune NxT Flow Cytometer (ThermoFisher Scientific)
Software	Data were collected using Attune NxT Software (ThermoFisher Scientific, version 2.7) and analyzed using FlowJo (Becton Dickinson, version 10.6).
Cell population abundance	This is not applicable. No cell sorting was performed in the present study
Gating strategy	All samples were initially gated using forward scatter and side scatter to identify events corresponding to lymphocytes, and then using viability dye to gate live cells. CD3 vs CD56 were used to gate target cells (CD4 cells) and effector cells (NK). From the CD3+ population, HIV-Gag vs CD4 were used for identifying the percentage of target cells that survived killing. The entirety of the gating for all of the samples studied is presented in Supplementary Fig. 5.

- [x] Tick this box to confirm that a figure exemplifying the gating strategy is provided in the Supplementary Information.
